# Synthesis of the methyl ester of 17(*R*/*S*)-Me-RvD5_n-3 DPA_ and relief of postoperative pain in male mice†

**DOI:** 10.1039/d4ob01534g

**Published:** 2024-10-30

**Authors:** Karina Ervik, Yi-ze Li, Ru-Rong Ji, Charles N. Serhan, Trond V. Hansen

**Affiliations:** a Department of Pharmacy, Section for Pharmaceutical Chemistry, University of Oslo P.O Box 1068 0316 Oslo Norway karina.ervik@farmasi.uio.no; b Center for Translational Pain Medicine, Department of Anesthesiology, Duke University Medical Center Durham North Carolina NC 27710 USA; c Center for Experimental Therapeutics and Reperfusion Injury, Department of Anesthesiology, Perioperative and Pain Medicine, Hale Building for Transformative Medicine, Brigham and Women's Hospital and Harvard Medical School Boston Massachusetts 02115 USA

## Abstract

The synthesis and biological evaluation of 17(*R*/*S*)-Me-RvD5_n-3 DPA_, an analog of the specialized pro-resolving mediators RvD5 and RvD5_n-3 DPA_, are presented. The synthesis was successfully accomplished utilizing Midland Alpine borane reduction, Sonogashira cross-coupling and a one-pot hydrozirconation/iodination protocol. *In vivo* evaluation of RvD5, RvD5_n-3 DPA_ and 17(*R*/*S*)-Me-RvD5_n-3 DPA_ in a mouse model of fracture revealed that all three compounds inhibited postoperative pain in male mice, but not in female mice.

## Abstract 2

The synthesis and biological evaluations of 17(*R*/*S*)-Me-RvD5_n-3 DPA_, an analog of the specialized pro-resolving mediator RvD5_n-3 DPA_, are presented.

## Graphical abstract

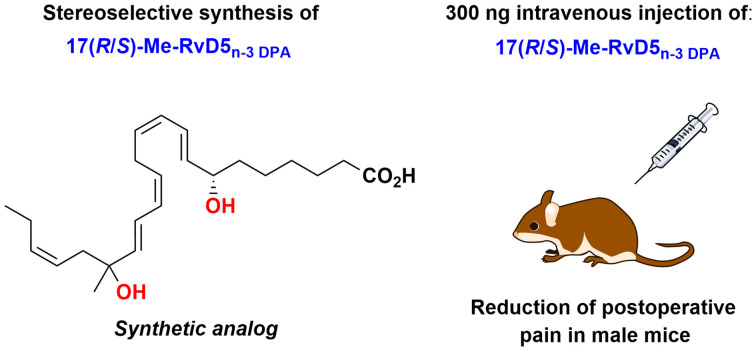

## Introduction

Acute inflammation is a response triggered by injury or pathogen invasion.^1^ The inflammatory response is crucial for defeating the intruding pathogen and for further tissue repair.^2^ Whenever the danger is over, the inflammation resolves and the body returns to homeostasis, referred to as the resolution phase.^3^ Failure in the resolution of inflammation may result in chronic inflammation, which in turn can lead to severe inflammatory diseases.^4^ The resolution phase is an active process where endogenously formed specialized pro-resolving mediators (SPMs) are central. SPMs are oxygenated polyunsaturated fatty acids (PUFAs), biosynthesized from the ω-6 PUFA arachidonic acid, and the ω-3 PUFAs eicosapentaenoic acid (EPA), docosahexaenoic acid (DHA), and n-3 docosapentaenoic acid (n-3 DPA).^5^ The SPMs are potent ligand agonists for G-protein coupled receptors (GPCRs), where activation leads to anti-inflammatory and pro-resolving actions,^6^ including reduction of inflammatory and neuropathic pain.^7,8^ Today, several individual classes of SPMs are known, such as lipoxins,^9,10^ resolvins,^11,12^ maresins,^13^ protectins,^11,14^ and the n-3 DPA series of resolvins, maresins and protectins.^15–17^

SPMs, endogenously formed during various inflammatory conditions, are non-toxic and well tolerated without immunosuppressive effects.^13,18^ Hence, SPMs have gained considerable interest in drug discovery projects.^6^ However, SPMs are rapidly locally metabolized by enzymes into less active products.^19^ For this reason, minor structural modifications on SPMs might prolong the time before enzymatic inactivation. However, such changes to the structure can also affect the potency of the molecule. Therefore, it is important that synthetic analogs contain the crucial pharmacophore as the native SPM, retaining the beneficial biological effects.^20,21^

**Fig. 1:**
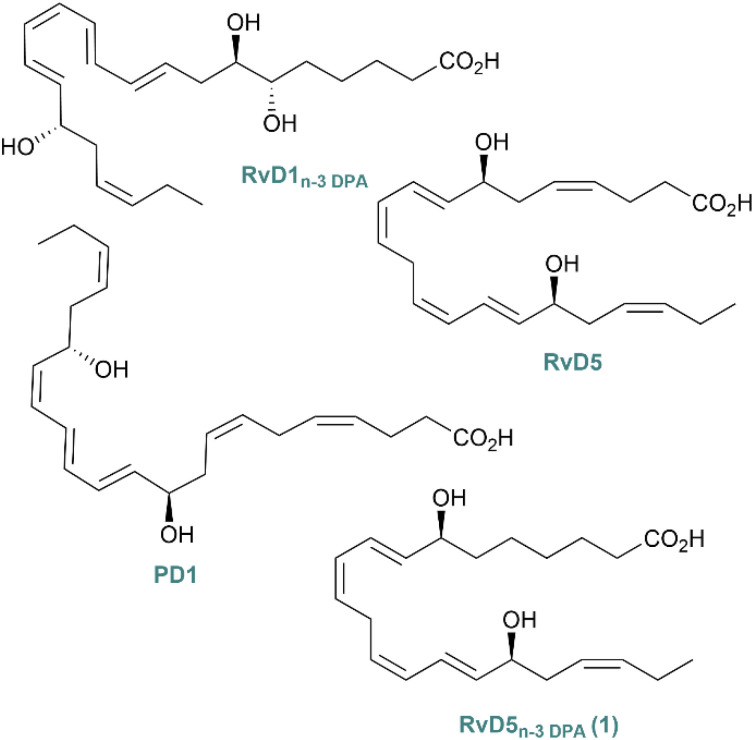
An overview of some SPMs.

Recently, RvD5_n-3 DPA_ (1) was stereoselectively synthesized and matched against the endogenously isolated 1 (Fig. 1).^22^ The SPM 1 displays potent pro-resolving effects upregulating the phagocytosis of bacteria by neutrophils and macrophages *via* the activation of the orphan receptor GPR101.^22,23^ The resolvin 1 is also linked with protective actions during rheumatoid arthritis, reducing joint inflammation.^24^ The congener of 1, RvD5 (Fig. 1), has shown a reduction in inflammatory and neuropathic pain in male mice, but not in female mice.^25^ This is the only example of a SPM that shows sex dimorphism in pain regulation. Since RvD5 and 1 are congeners, it was of interest to find out if 1 shared the same properties, knowing they both are agonists for GPR101.^23^ Protectin D1 (PD1), also called neuroprotectin D1 when isolated in neural cells, has proved to alleviate neuropathic pain in mouse models.^21^ Studies of the GPR101 receptor in mice and humans uncovered its expression in brain regions, such as the hypothalamus and amygdala.^26,27^ Knowing that the receptor recognising 1 is expressed in the brain, it was of interest to investigate pain effects in mice, since effective non-opioid pain treatments are needed, which constitutes a public health crisis.^8^

**Scheme 1:**
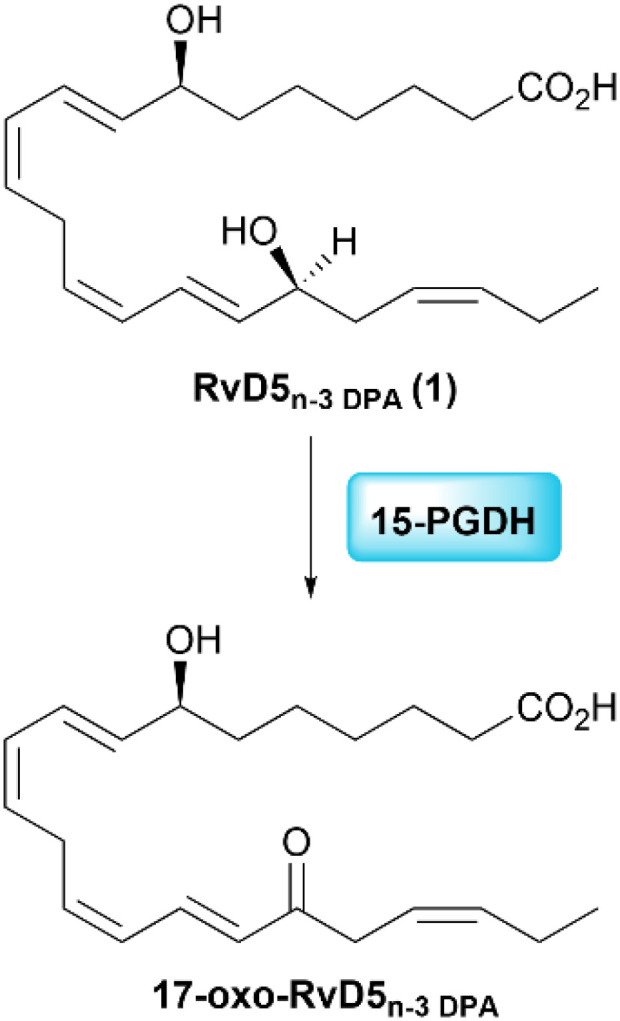
Enzymatic inactivation of RvD5_n-3 DPA_ (1) by 15-PGDH.

One of the metabolic pathways observed for RvD5_n-3 DPA_ is *via* the enzyme 15-prostaglandin dehydrogenase (15-PGDH), yielding the inactive product 17-oxo-RvD5_n-3 DPA_ (Scheme 1).^24^ By replacing the hydrogen at C17 with a methyl group, 15-PGDH oxidation to the 17-oxo metabolite is anticipated to be diminished, as reported for other SPM analogs.^28–30^

**Scheme 2:**
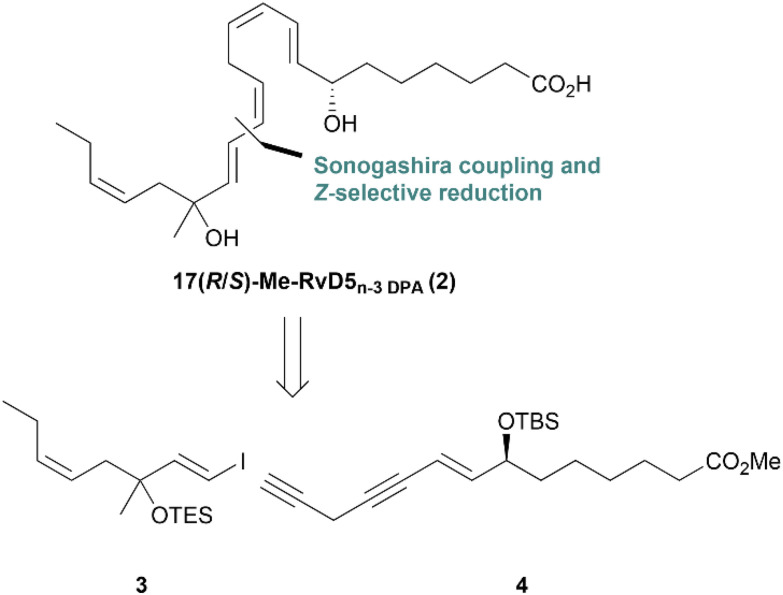
Retrosynthetic analysis of the analog 2.

Against the information provided, it was of interest to synthesize the analog mimetic named 17(*R*/*S*)-Me-RvD5_n-3 DPA_ (2) based on the retrosynthetic analysis of 2 (Scheme 2), where compounds 3 and 4 were identified.

## Results and discussion

**Scheme 3:**
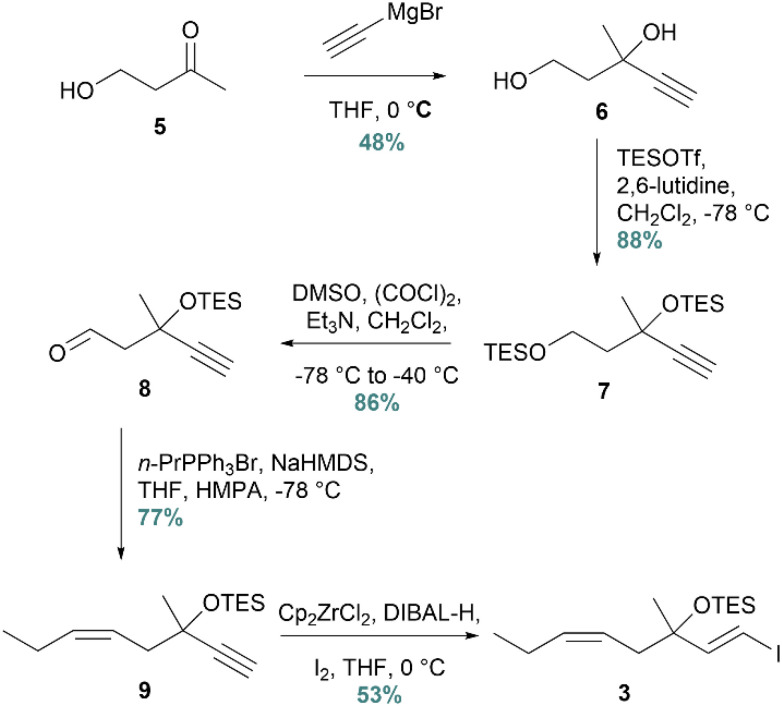
Synthesis of vinyl iodide 3.

The synthesis was initiated with the preparation of vinylic iodide 3 from commercially available 4-hydroxybutan-2-one (5) (Scheme 3). A Grignard reaction was successfully conducted with ethynylmagnesium bromide, affording diol 6 in 48% yield. In the next step, bis-TES-protection gave compound 7, while selective deprotection of the primary TES-group and oxidation afforded the corresponding aldehyde 8. This one-pot Swern oxidation protocol required strict temperature control.^31^ The resulting aldehyde was then subjected to a *Z*-selective Wittig reaction, yielding alkyne 9 in 77%. Lastly, vinyl iodide 3 was afforded in 53% yield after an *E*-selective hydrozirconation/iodination protocol.

**Scheme 4:**
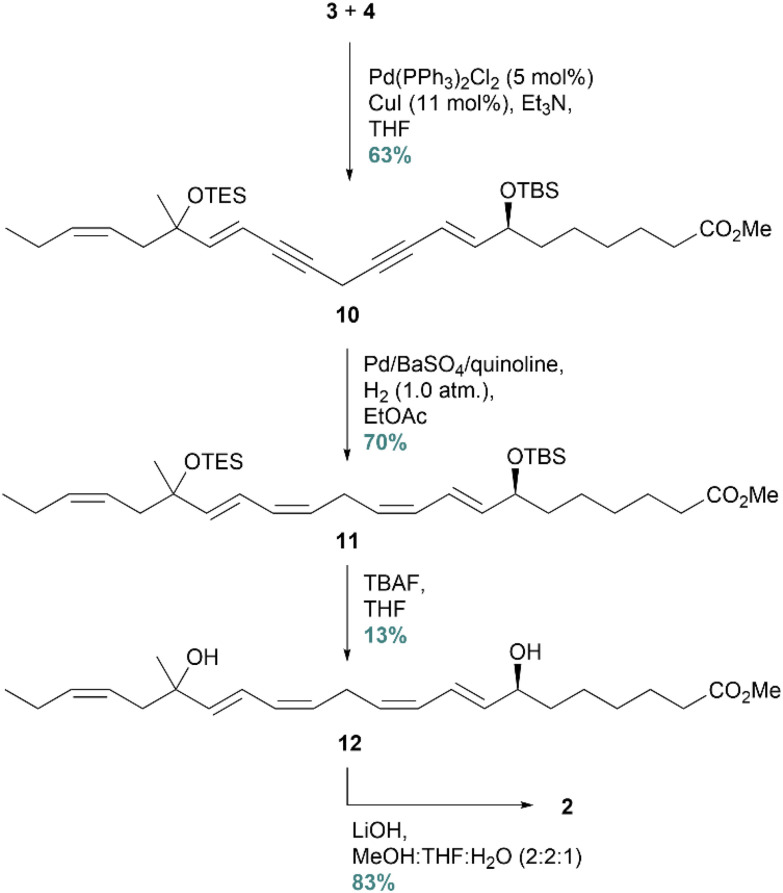
Sonogashira cross-coupling followed by *Z*-selective reduction yielding the desired product 2.

The labile terminal alkyne 4 was resynthesized based on a protocol developed in our group,^22^ in order to proceed with the synthesis of 2. The complete carbon skeleton was constructed by a Sonogashira cross-coupling reaction, yielding 10 in 63% isolated yield (Scheme 4). Next, the diyne was *Z*-selectively reduced to produce 11 by utilizing the Rosenmund hydrogenation protocol. This protocol was successfully implemented in the synthesis of RvD5_n-3 DPA_, because the milder hydrogenation reducing agent Lindlar's catalyst yielded no product. In the next step, the protecting groups were removed in the presence of TBAF. Surprisingly, the crude reaction mixture contained impurities that turned out to be challenging to remove by column chromatography. To achieve a chemical purity of 12 > 96% (HPLC analyses), it became necessary to purify the compound by preparative TLC. For this reason, the isolated yield became disappointingly low, only 13%. There are examples of deprotection of silyl ethers by *in situ* generated HCl to avoid the formation of byproducts.^32^ However, this method was not suitable in our case due to the labile C17 tertiary alcohol.

The analog 17(*R*/*S*)-Me-RvD5_n-3 DPA_ (2) was stored as its methyl ester 12. Just upon biological testing, this ester was hydrolysed, yielding the target compound 2 in 83%. Spectral data (NMR, UV, and MS) agreed with the structure of 2 (ESI data†). The SPM 1, as mentioned, was recently stereoselectively synthesized and matched using LC/MS-MS MRM and UV experiments,^22^ while RvD5 is now commercially available.

**Fig. 2:**
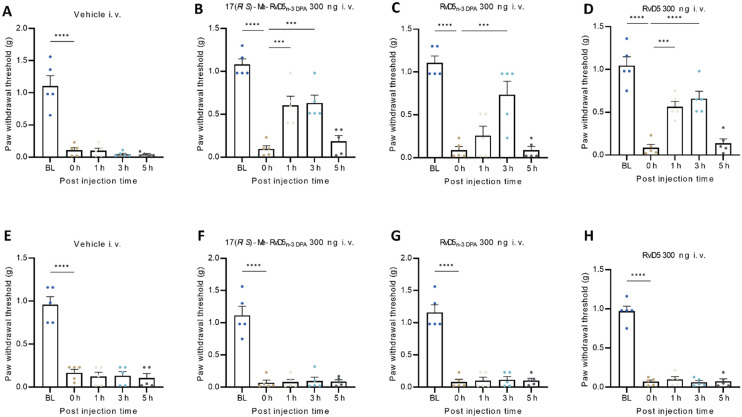
Intravenous treatment of RvD5, RvD5_n-3 DPA_ (1) and analog 17(*R*/*S*)-Me-RvD5_n-3 DPA_ (2) reduces fracture induced mechanical pain in male mice but not in female mice. von Frey testing showing PWTs and the pain-relieving effects of 17(*R*/*S*)-Me-RvD5_n-3 DPA_, RvD5_n-3 DPA_, and RvD5 (i.v., 300 ng) in the fracture model in male mice (A–D) but not in female mice (E–H). The drugs and vehicle were given two days after fracture surgery. *n* = five animals per sex per group, ***p = 0.001, ****p = 0.0001, one-way ANOVA followed by Bonferroni *post-hoc* comparison. The data are presented as mean ± SEM.

We compared the analgesic effects of 17(*R*/*S*)-Me-RvD5_n-3 DPA_, RvD5_n-3 DPA_, and RvD5 with vehicle (PBS) treatment by intravenous (i.v.) administration of each compound at a dose of 300 ng in 100 μl of PBS solution two days after bone fracture. The paw withdrawal thresholds (PWTs) were assessed before surgery as the baseline (BL), after the surgery and prior to the drug treatment (0 h), and then at 1 h, 3 h, and 5 h after the treatment (Fig. 2A–H). The fracture resulted in a substantial reduction in the PWT at 0 h. Vehicle treatment had no effects on PWTs at all the time points examined in males (Fig. 2A) and females (Fig. 2E). Notably, i.v. administration of either 17(*R*/*S*)-Me-RvD5_n-3 DPA_, RvD5_n-3 DPA_, or RvD5 significantly increased PWTs in male mice, compared to 0 h (Fig. 2A–D). 17(*R*/*S*)-Me-RvD5_n-3 DPA_ and RvD5 increased the PWT both at 1 h and 3 h but not at 5 h. RvD5_n-3 DPA_ (1) only increased the PWT at 3 h (Fig. 2A–D). These results indicate that 17(*R*/*S*)-Me RvD5_n-3 DPA_ and RvD5 are more effective than RvD5_n-3 DPA_ in reducing mechanical pain. Strikingly, none of the compounds showed any pain-relieving effects in female mice (Fig. 2E–H). These results strongly suggest that RvD5, RvD5_n-3 DPA_ (1) and 17(*R*/*S*)-Me-RvD5_n-3 DPA_ (2) reduced mechanical pain only in males in this postoperative pain model.

## Conclusions

To summarize, the synthesis of the methyl ester of 17(*R*/*S*)-Me-RvD5_n-3 DPA_ (2) was successfully accomplished in 1.2% yield over 13 steps (longest linear sequence). The novel synthetic SPM analog 17(*R*/*S*)-Me-RvD5_n-3 DPA_ (2) showed great potential to reduce postoperative pain in a sex-dependent manner compared to RvD5_n-3 DPA_ (1). It has been reported that n-3 DPA derived SPMs show reduced ability to ameliorate neuropathic pain over time,^8,21^ and herein we report that the SPM 1 also shows diminished effects at 5 h, perhaps due to the rapid enzymatic β-oxidation processes of 1, as reported for protectin D1,^33^ compared to RvD5 and analog 2. Moreover, new knowledge on the structure–function relationships and biomolecular properties mediated by the compounds investigated herein towards the receptor GPR101 is in demand. These results will be reported in due time.

## Author contributions

K. E.: writing original draft, editing, methodology, investigation, and analysis. Y.-Z. L.: writing, methodology, investigation, and analysis. R.-R. J.: conceptualization, supervision, and methodology. C. N. S.: methodology and conceptualization. T. V. H.: conceptualization, editing, and supervision.

## Data availability

Synthetic and biological experiment protocols together with characterization data (NMR, HRMS, HPLC, and UV-vis) are available in the ESI file.†

## Conflicts of interest

There are no conflicts to declare.

## Supplementary Material

OB-022-D4OB01534G-s001
