# Supplementary material for: Synthesis of the methyl ester of 17(R/S)-Me-RvD5n-3 DPA and relief of postoperative pain in male mice
Source: Org Biomol Chem. 2024 Oct 30;22(47):9266–70. doi: 10.1039/d4ob01534g (PMC11563200; doi:10.1039/d4ob01534g)
Supplement: OB-022-D4OB01534G-s001 [file OB-022-D4OB01534G-s001.pdf]

Supplementary Information (SI) for Organic & Biomolecular Chemistry.
This journal is © The Royal Society of Chemistry 2024

Supporting information for Synthesis of methyl ester of 17(*R*/*S*)-Me-RvD5$_{n\text{-}3\ DPA}$ and relief of postoperative pain in male mice

Karina Ervik,*[,a] Yi-ze Li,[b] Ru-Rong Ji,[b] Charles N. Serhan,[c] and Trond V. Hansen[a]

[a] *Department of Pharmacy, Section for Pharmaceutical Chemistry, University of Oslo, PO Box 1068, N-0316, Oslo, Norway*

[b] *Center for Translational Pain Medicine, Department of Anesthesiology, Duke University Medical Center, Durham, North Carolina, NC 27710, USA*

[c] *Center for Experimental Therapeutics and Reperfusion Injury, Department of Anesthesiology, Perioperative and Pain Medicine, Hale Building for Transformative Medicine, Brigham and Women's Hospital and Harvard Medical School, Boston, Massachusetts, 02115*

Table of Contents

General Information 2
Synthetic protocols 3
Biological experiments in animals 7
NMR Spectra 8
HRMS Spectra 17
HPLC chromatogram 22
UV-Vis spectra 23
References 25

General Information

Unless otherwise stated, all commercially available reagents and solvents were used in the form they were supplied without any further purification. The stated yields are based on the isolated material. All sensitive reactions were performed under an argon atmosphere using Schlenk techniques. Reaction flasks were covered with aluminum foil during sensitive reactions and storage to minimize exposure to light. Thin layer chromatography was performed on silica gel 60 F254 aluminum-backed plates fabricated by Merck. Flash column chromatography was performed on silica gel 60 (40 - 63 µm) fabricated by Merck. NMR spectra were recorded on a Bruker AVII 400, AVII 600 or AVIII 800 spectrometer at 400 MHz/600 MHz/800 MHz for ^{1}H NMR and at 101 MHz/151 MHz/201 MHz for ^{13}C NMR. Coupling constants (*J*) are reported in hertz and chemical shifts are reported in parts per million (δ) relative to the central residual protium solvent resonance in ^{1}H NMR ($CDCl_3$ = δ 7.26 and CD_3OD = δ 3.31) and the central carbon solvent resonance in ^{13}C NMR ($CDCl_3$ = δ 77.16 ppm and CD_3OD = δ 49.0). High-resolution mass spectra were recorded at 70 eV on a Micromass Prospec Q or Micromass QTOF 2W spectrometer using ESI as the method of ionization. Optical rotations were measured using a 0.2 mL cell with a 0.1 dm path length on a PerkinElmer 341 polarimeter. HPLC-analyses were performed using a C18 stationary phase (Eclipse XDBC18, 4.6 x 250 mm, particle size 5 µm, from Agilent Technologies), applying the conditions stated. The UV-Vis spectrum was recorded using an Agilent Technologies Cary 8485 UV-Vis spectrophotometer using quartz cuvettes.

Synthetic protocols

(±)-3-Methylpent-4-yne-1,3-diol (6)

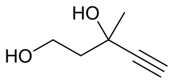

4-Hydroxybutan-2-one **5** (2.00 g, 1.96 mL, 22.7 mmol, 1.00 equiv.) was dissolved in dry THF (100 mL) and cooled to 0 °C. Ethynylmagnesium bromide (0.5 M in THF, 91.0 mL, 45.5 mmol, 2.00 equiv.) was added in a dropwise manner. The reaction mixture was allowed to attain ambient temperature overnight. Then, the reaction mixture was re-cooled to 0 °C and carefully treated with saturated aqueous NaH_2PO_4. The phases were separated and the aqueous phase was extracted with Et_2O (3 × 30 mL). The combined organic phases were dried over Na_2SO_4, filtered and concentrated *in vacuo*. The residue was purified by column chromatography on silica gel (heptane, followed by 50% EtOAc in heptane). This afforded the title compound as a colorless liquid in 48% yield (1.233 g, 10.90 mmol). Spectroscopic data were in agreement with those reported in the literature.[1] R_f (50% EtOAc in heptane, visualized by $KMnO_4$ stain) = 0.17; ^{1}H NMR (400 MHz, $CDCl_3$) δ 4.20 – 4.12 (m, 1H), 3.94 – 3.87 (m, 1H), 3.49 – 3.30 (br, 2H), 2.49 (s, 1H), 2.03 – 1.94 (m, 1H), 1.85 – 1.78 (m, 1H), 1.53 (s, 3H); ^{13}C NMR (101 MHz, $CDCl_3$) δ 87.08, 72.09, 68.76, 60.68, 43.40, 30.70.

(±)-1,3-Bis(triethylsilyl(oxy))3-methylpent-4-yn (7)

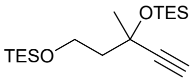

(±)-3-Methylpent-4-yne-1,3-diol **6** (250 mg, 2.19 mmol, 1.00 equiv.) was dissolved in CH_2Cl_2 (25 mL) and cooled to -78 °C. 2,6-Lutidine (1.41 g, 1.52 mL, 13.1 mmol, 6.00 equiv.) was added, followed by TESOTf (1.27 g, 1.09 mL, 4.82 mmol, 2.20 equiv.). The reaction mixture was allowed to attain ambient temperature overnight. After 24h *in toto*, the reaction mixture was treated with saturated aqueous NH_4Cl (25 mL) and CH_2Cl_2 (25 mL). The phases were separated and the aqueous phase was extracted with CH_2Cl_2 (3 × 20 mL). The combined organic phases were dried over Na_2SO_4, filtered and the solvent was evaporated *in vacuo*. The residue was purified by column chromatography on silica gel (heptane, followed by 5% Et_2O in heptane). This afforded the title compound as colorless liquid in 88% yield (660 mg, 1.93 mmol). R_f (5% Et_2O in heptane, visualized by CAM stain) = 0.56; ^{1}H NMR (400 MHz, $CDCl_3$) δ 3.88 – 3.80 (m, 2H), 2.40 (s, 1H), 2.00 – 1.86 (m, 2H), 1.47 (s, 3H), 0.99 – 0.93 (m, 18H), 0.69 – 0.57 (m, 12H); ^{13}C NMR (151 MHz, $CDCl_3$) δ 87.96, 77.37, 77.16, 76.95, 72.05, 67.61, 59.70, 47.77, 31.56, 7.09 (3C), 6.91 (3C), 6.23 (3C), 4.62 (3C).

3-Methyl-3-((triethylsilyl)oxy)pent-4-ynal (8)

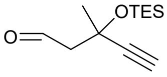

Oxalyl chloride (2 M in CH_2Cl_2, 2.79 mL, 5.57 mmol, 4.40 equiv.) was added dropwise to a solution of DMSO (0.78 mL, 11.0 mmol, 8.70 equiv.) in dry CH_2Cl_2 (5 mL) at -78 °C. The solution was stirred for 20 min before (±)-1,3-bis(triethylsilyl(oxy))3-methylpent-4-yn **7** (433 mg, 1.27 mmol, 1.00 equiv.) dissolved in CH_2Cl_2 (4 mL) was added in a dropwise manner. The reaction was stirred for 45 min at -78 °C, thereafter 55 min at -40 °C before it was re-cooled to -78 °C. Triethylamine (2.64 mL, 19.1 mmol,

15.0 equiv.) was added, and the reaction mixture stirred for additional 10 min before it was allowed to reach ambient temperature. H_2O (10 mL) was added before the mixture was diluted with CH_2Cl_2 (15 mL). The phases were separated, and the aqueous phase was extracted with CH_2Cl_2 (3 × 10 mL). The organic phases were combined, dried over Na_2SO_4, filtered, and concentrated *in vacuo*. The resulting residue was purified by column chromatography on silica gel (5% Et_2O in heptane) to afford aldehyde **8** as a colorless oil in 86% yield (246 mg, 1.09 mmol). The aldehyde was used immediately in the next reaction. R_f (10% EtOAc in heptane, visualized by CAM and $KMnO_4$ stain) = 0.29; ^1H NMR (400 MHz, $CDCl_3$) δ 9.88 (t, *J* = 2.8 Hz, 1H), 2.62 (d, *J* = 2.8 Hz, 2H), 2.57 (s, 1H), 1.58 (s, 3H), 0.96 (t, *J* = 7.9 Hz, 9H), 0.76 – 0.62 (m, 6H). ^{13}C NMR (151 MHz, $CDCl_3$) δ 201.69, 86.59, 73.87, 66.40, 57.28, 31.55, 7.01 (3C), 6.12 (3C).

(*Z*)-Triethyl((3-methyloct-5-en-1-yn-3-yl)oxy)silane (9)

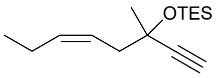

Propyltriphenylphosphonuim bromide (336 mg, 0.87 mmol, 1.20 equiv.) was suspended in dry THF (7.0 mL) and HMPA (1.5 mL). The suspension was degassed twice (switching from vacuum and Ar-atmosphere) before cooled to -78 °C. NaHMDS (0.6 M in toluene, 1.45 mL, 0.87 mmol, 1.20 equiv.) was added dropwise, giving the suspension a slightly yellow color. The cooling bath was removed, allowing the suspension to reach rt. When the color changed to red/orange (ca. 30 min), it was re-cooled to -78 °C. Aldehyde **8** (165 mg, 0.73 mmol, 1.00 equiv.) was azeotroped with 2-MeTHF (2 × 1 mL) before dissolved in dry THF (2.0 mL) and added dropwise at -78 °C. The reaction was allowed to reach ambient temperature, and stirred until deemed completed by TLC (~2 hours). The reaction was quenched with phosphate buffer (pH = 7, 4 mL) and H_2O (7 mL), and diluted with Et_2O (7 mL). The aqueous phase was extracted with Et_2O (2 × 10 mL) before the organic phases were combined, dried over Na_2SO_4, filtered and concentrated *in vacuo*. The residue was purified by column chromatography on silica gel (2.5% Et_2O in heptane) to afford the product **9** (142 mg, 0.56 mmol) as a colorless oil in 77% yield. R_f (5% EtOAc in heptane, visualized by $KMnO_4$ stain) = 0.65; ^1H NMR (400 MHz, $CDCl_3$) δ 5.57 – 5.44 (m, 2H), 2.48 – 2.34 (m, 3H), 2.11 – 2.02 (m, 2H), 1.43 (s, 3H), 0.97 (t, *J* = 7.8 Hz, 12H), 0.73 – 0.63 (m, 6H). ^{13}C NMR (101 MHz, $CDCl_3$) δ 134.36, 124.06, 88.44, 71.96, 68.94, 42.83, 30.54, 20.98, 14.35, 7.15 (3C), 6.24 (3C); HRESIMS *m/z*: 275.1802 $[M + Na]^+$ (calcd for $C_{15}H_{28}OSiNa$, 275.1802).

Triethyl(((1*E*,5*Z*)-1-iodo-3-methylocta-1,5-dien-3-yl)oxy)silane (3)

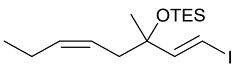

To a suspension of bis(cyclopentadienyl)zirconium(IV) dichloride (643 mg, 2.12 mmol, 2.00 equiv.) in THF (2.5 mL) was added DiBAl-H (1.0 M in THF, 1.90 mL, 1.90 mmol, 1.80 equiv.) at 0 °C. After stirring for 30 min, a solution of alkyne **9** (269 mg, 1.06 mmol, 1.00 equiv.) in THF (0.5 mL) was added, and the suspension stirred for an additional 60 min, allowing to reach ambient temperature. A solution of iodine (350 mg, 1.38 mmol, 1.30 equiv.) in THF (0.5 mL) was added at rt to the yellow solution and stirring was continued for further 40 min. The reaction was quenched by addition of sat. aq. $Na_2S_2O_3$ (1.0 mL), H_2O (0.8 mL) and sat. aq. $NaHCO_3$ (1.0 mL). The mixture was stirred for 5 min before the phases were separated, and the aqueous phase was extracted with CH_2Cl_2 (3 × 5 mL). The combined organic phases were dried ($MgSO_4$), filtrated, and concentrated *in vacuo*. The crude product thus

obtained was purified by column chromatography (SiO_2, heptane) to obtain the vinyl iodide **3** (213 mg, 0.56 mmol) as a colorless oil in 53% yield. R_f (heptane, visualized by UV and $KMnO_4$ stain) = 0.22; ^1H NMR (400 MHz, $CDCl_3$) δ 6.56 (d, *J* = 14.4 Hz, 1H), 6.21 (d, *J* = 14.4 Hz, 1H), 5.52 – 5.42 (m, 1H), 5.39 – 5.30 (m, 1H), 2.24 (d, *J* = 7.3 Hz, 2H), 2.07 – 1.96 (m, 2H), 1.28 (s, 3H), 0.98 – 0.92 (m, 12H), 0.62 – 0.55 (m, 6H). ^{13}C NMR (101 MHz, $CDCl_3$) δ 152.79, 134.27, 123.82, 78.18, 75.25, 41.28, 26.72, 20.92, 14.31, 7.18 (3C), 6.75 (3C); HRESIMS *m/z*: 403.0924 $[M + Na]^+$ (calcd for $C_{15}H_{29}IOSiNa$, 403.0925).

Methyl (7*S*,8*E*,15*E*,19*Z*)-7-((*tert*-butyldimethylsilyl)oxy)-17-methyl-17-((triethylsilyl)oxy)docosa-8,15,19-trien-10,13-diynoate (10)

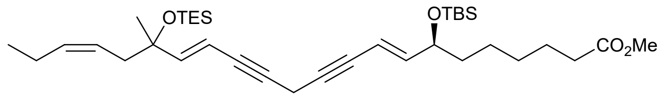

Vinyl iodide **3** (25 mg, 0.07 mmol, 1.00 equiv.) was dissolved in THF (0.8 mL), and the solution was cooled to 0 °C before $Pd(PPh_3)_2Cl_2$ (2.5 mg, 3.5 µmol, 5.0 mol %), CuI (1.5 mg, 0.8 µmol, 11 mol %), and Et_3N (13 mg, 18 µL, 0.13 mmol, 2.0 equiv.) were added. Alkyne **4** (42 mg, 0.13 mmol, 2.0 equiv.) was dissolved in THF (0.3 mL) and added dropwise. The reaction mixture was allowed to slowly warm up to rt and stirred in the dark for 16 h. After completion, the reaction mixture was filtrated through a plug of silica gel (15% EtOAc in heptane) and concentrated *in vacuo*. The crude product thus obtained was purified by column chromatography (SiO_2, 5% EtOAc in heptane) to obtain product **10** (27 mg, 0.04 mmol) as a pale yellow oil in 63% yield. R_f (10% EtOAc in heptane, visualized by UV and $KMnO_4$ stain) = 0.32; $[\alpha]_D^{20}$ = -5.00 (c 0.2, CH_2Cl_2); ^1H NMR (400 MHz, $CDCl_3$) δ 6.16 – 6.03 (m, 2H), 5.69 – 5.57 (m, 2H), 5.49 – 5.40 (m, 1H), 5.38 – 5.29 (m, 1H), 4.16 – 4.09 (m, 1H), 3.66 (s, 3H), 3.42 (s, 2H), 2.33 – 2.22 (m, 4H), 2.05 – 1.96 (m, 2H), 1.66 – 1.57 (m, 2H), 1.50 – 1.43 (m, 2H), 1.32 – 1.26 (m, 7H), 0.95 (td, *J* = 7.7, 2.1 Hz, 12H), 0.89 (s, 9H), 0.58 (q, *J* = 7.9 Hz, 6H), 0.03 (s, 3H), 0.02 (s, 3H). ^{13}C NMR (101 MHz, $CDCl_3$) δ 174.35, 150.31, 146.51, 133.93, 124.18, 108.57, 107.23, 83.67, 83.50, 79.35, 79.10, 77.48, 77.16, 76.84, 75.43, 72.52, 51.61, 41.50, 37.80, 34.17, 29.29, 27.28, 25.99 (3C), 25.04, 24.69, 20.94, 18.34, 14.22, 11.29, 7.23 (3C), 6.87 (3C), -4.33, -4.72; HRESIMS *m/z*: 637.4081 $[M + Na]^+$ (calcd for $C_{36}H_{62}O_4Si_2Na$, 637.4079).

Methyl (7*S*,8*E*,10*Z*,13*Z*,15*E*,19*Z*)-7-((*tert*-butyldimethylsilyl)oxy)-17-methyl-17-((triethylsilyl)oxy)docosa-8,10,13,15,19-pentaenoate (11)

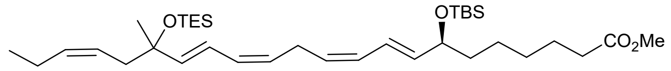

Diyne **10** (20 mg, 33 µmol, 1.0 equiv.) was dissolved in EtOAc (2.0 mL) under argon. Quinoline (20 µL, 0.24 mmol) and 5% Pd/$BaSO_4$ (20 mg) was added before the flask was evacuated and refilled with hydrogen gas twice. The reaction was stirred for 1 h before it was filtrated through a short plug of silica gel (15% EtOAc in heptane) and concentrated *in vacuo*. The crude product thus obtained was purified by column chromatography (SiO_2, 5% EtOAc in heptane) to obtain product **11** (14 mg, 23 µmol) as a colorless oil in 70% yield. R_f (10% EtOAc in heptane, visualized by UV and $KMnO_4$ stain) = 0.37; $[\alpha]_D^{20}$ = +5.20 (c 0.2, CH_2Cl_2); ^1H NMR (400 MHz, $CDCl_3$) δ 6.52 – 6.36 (m, 2H), 6.08 – 5.91 (m, 2H), 5.74 (d, *J* = 15.3 Hz, 1H), 5.65 (dd, *J* = 15.1, 6.3 Hz, 1H), 5.48 – 5.30 (m, 4H), 4.15 (q, *J* = 5.9 Hz, 1H), 3.66 (s, 3H), 3.06 (t, *J* = 7.5 Hz, 2H), 2.33 – 2.22 (m, 4H), 2.07 – 1.97 (m, 2H), 1.67 – 1.58 (m, 2H), 1.52 – 1.42 (m, 2H), 1.32 – 1.26 (m, 7H), 0.98 – 0.91 (m, 12H), 0.90 – 0.88 (m, 9H), 0.58 (q, *J* = 7.9 Hz, 6H), 0.05 (s, 3H), 0.03

(s, 3H). ^{13}C NMR (101 MHz, $CDCl_3$) δ 174.39, 141.56, 137.72, 133.58, 129.15, 129.06, 128.95, 128.67, 124.76, 124.25, 122.51, 77.48, 77.16, 76.84, 75.34, 73.25, 51.60, 41.95, 38.34, 34.21, 32.03, 29.32, 27.28, 26.60, 26.04 (3C), 25.09, 20.93, 18.40, 14.27, 7.26 (3C), 6.91 (3C), -4.12, -4.62; HRESIMS *m/z*: 641.4390 $[M + Na]^+$ (calcd for $C_{36}H_{66}O_4Si_2Na$, 641.4392).

Methyl (7*S*,8*E*,10*Z*,13*Z*,15*E*,19*Z*)-7,17-dihydroxy-17-methyldocosa-8,10,13,15,19-pentaenoate (12)

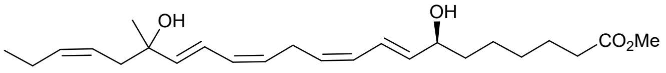

The bis-silyl protected compound **11** (5.00 mg, 0.003 mmol, 1.00 equiv.) was dissolved in 0.2 mL dry THF and cooled to 0 °C. TBAF (1 M in THF, 0.01 mL, 0.01 mmol, 5.00 equiv.), was added and the reaction mixture stirred overnight, allowing to reach rt. The reaction was quenched with phosphate buffer (pH = 7, 0.1 mL), brine (0.2 mL) and diluted with CH_2Cl_2 (0.5 mL). The phases were separated, and the aqueous phase was extracted with CH_2Cl_2 (2 × 0.5 mL). The combined organic phases were dried over Na_2SO_4, filtered and concentrated *in vacuo*. The resulting residue was purified by column chromatography on silica gel (2.5% MeOH in CH_2Cl_2), thereafter on preparative TLC (2.5% MeOH in CH_2Cl_2) to afford the 17(*R*/*S*)-Me-RvD5$_{n\text{-}3\ DPA}$ methyl ester (0.5 mg, 0.39 µmol, 13%) as a colorless oil. The chemical purity (96%) was determined by HPLC analysis (Eclipse XDB-C18, MeOH/H_2O 76:24, 1.0 mL/min): t_r (minor) = 20.09 min, t_r (major) = 22.89 min. UV-Vis (MeOH): λ_{max} 243 nm (log ε = 4.64); R_f (40% EtOAc in heptane, visualized by UV and $KMnO_4$ stain) = 0.18; $[\alpha]_D^{20}$ = +4.10 (c 0.2, MeOH); ^1H NMR (600 MHz, MeOD-higher-lock-power) δ 6.61 – 6.53 (m, 2H), 6.00 (q, *J* = 10.9 Hz, 2H), 5.77 (d, *J* = 15.3 Hz, 1H), 5.67 (dd, *J* = 15.1, 6.7 Hz, 1H), 5.44 – 5.30 (m, 4H), 4.55 (s, 2H), 4.12 – 4.08 (m, 1H), 3.65 (s, 3H), 3.13 – 3.07 (m, 2H), 2.34 – 2.29 (m, 4H), 2.07 – 2.04 (m, 2H), 1.63 – 1.60 (m, 2H), 1.54 – 1.48 (m, 2H), 1.35 – 1.32 (m, 4H), 1.27 (s, 3H), 0.95 (t, *J* = 7.5 Hz, 3H). ^{13}C NMR (201 MHz, MeOD) δ 175.99, 141.95, 138.05, 134.92, 130.27, 129.82, 129.71, 129.59, 126.24, 125.13, 123.62, 73.72, 73.20, 51.95, 41.33, 38.22, 34.75, 33.07, 30.12, 27.40, 26.22, 25.98, 21.66, 14.48. HRESIMS *m/z*: 413.2662 $[M + Na]^+$ (calcd for $C_{24}H_{38}O_4Na$, 413.2662).

(7*S*,8*E*,10*Z*,13*Z*,15*E*,19*Z*)-7,17-Dihydroxy-17-methyldocosa-8,10,13,15,19-pentaenoic acid (2)

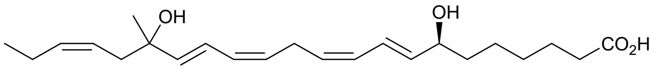

The methyl ester **12** (210 µg, 0.54 µmol, 1.00 equiv.) was dissolved in a solution of MeOH:THF:H_2O (2:2:1, 0.2 mL) and cooled to 0 °C before LiOH (1.0 mg, 0.04 mmol, 50 equiv.) was added in one portion. The reaction mixture was stirred for 4 h, allowing to reach ambient temperature before it was acidified by addition of sat. aq. NaH_2PO_4 (0.3 mL) and diluted with EtOAc (0.3 mL). The phases were separated, and the aqueous phase was extracted with EtOAc (3 × 0.5 mL). The combined organic phases were dried over $MgSO_4$ and directly filtered through a short plug of silica gel to yield the title compound **2** in 83 % (168 µg, 0.45 µmol) as a colorless oil. UV-Vis (EtOH): λ_{max} 243 nm (log ε = 4.64); R_f (5% MeOH in CH_2Cl_2, visualized by UV and $KMnO_4$ stain) = 0.19.

Biological experiments in animals

Animal protocols were approved by the Animal Care Committee of Duke University and we followed the ethics guidelines of the International Association for the Study of Pain. Adult CD1 mice (male and female, 25-35 grams) were obtained from Charles River Laboratories and housed in a light and humidity-controlled facility. Animals were habituated in the behavioural testing room for at least two days prior to baseline testing. Animals were randomly assigned to each experimental group. The mice were anesthetized with isoflurane prior to the procedure. The tibial fracture was performed as previously reported.[2] An incision was made on the left knee and a stainless-steel pin (0.38 mm) was inserted into the intramedullary canal, followed by osteotomy. The incision was closed with 4-0 Prolene sutures. Mice were anesthetized with isoflurane while receiving intravenous injection. For intravenous (i.v.) treatments, drugs were dissolved in PBS as vehicle (100 µL injection volume, 300 ng/mice) and injected into the tail vein. Mechanical pain was assessed in von Frey test conducted in a blinded manner, as previously reported. For each test, mice were placed in individual plastic boxes (5 cm × 5 cm) on an elevated mesh floor and habituated for at least 30 min. The paw withdrawal threshold (PWT) was determined via Dixon's up-down method using von Frey filaments with logarithmically increasing stiffnesses (0.02-2.56 g, Stoelting).[3, 4] The filaments were applied perpendicularly to the central plantar surface of the hind paw.

NMR Spectra

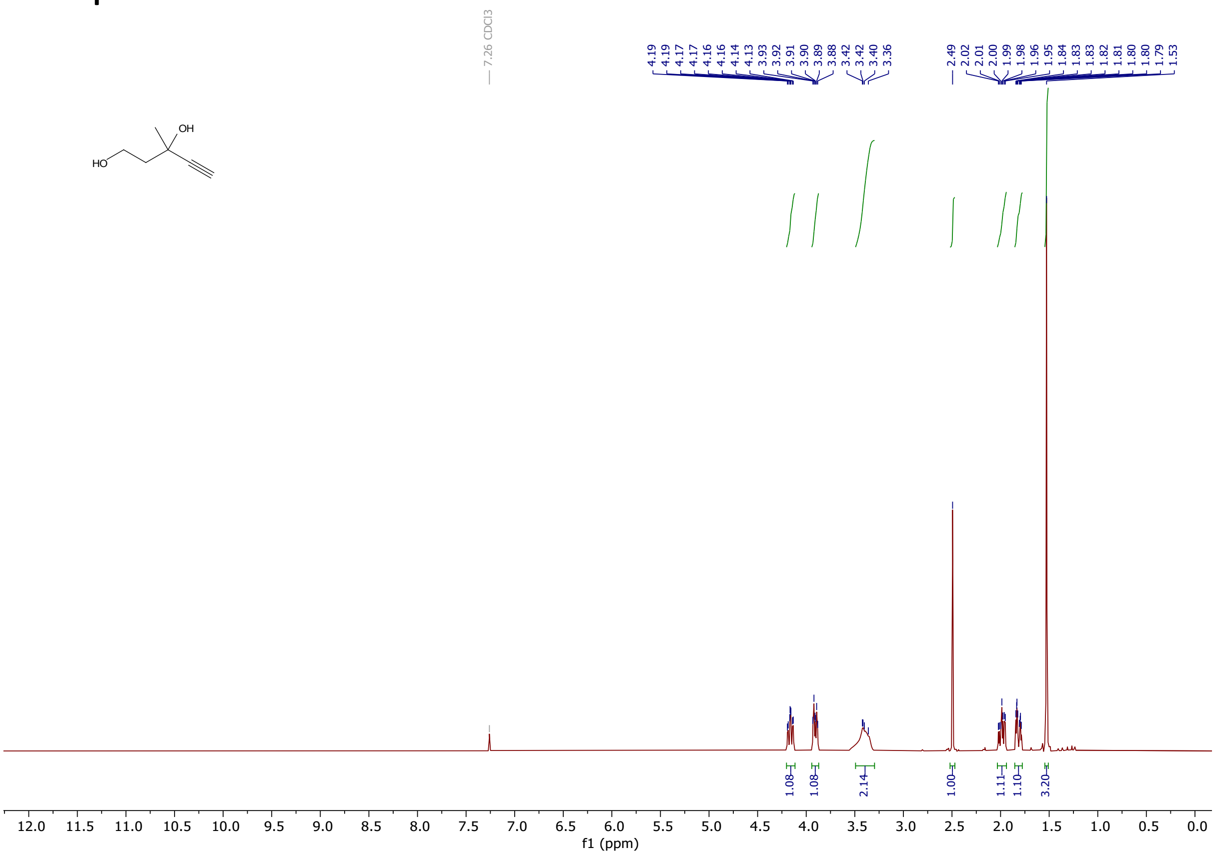

Figure 1: ^{1}H NMR spectrum of compound **6**.

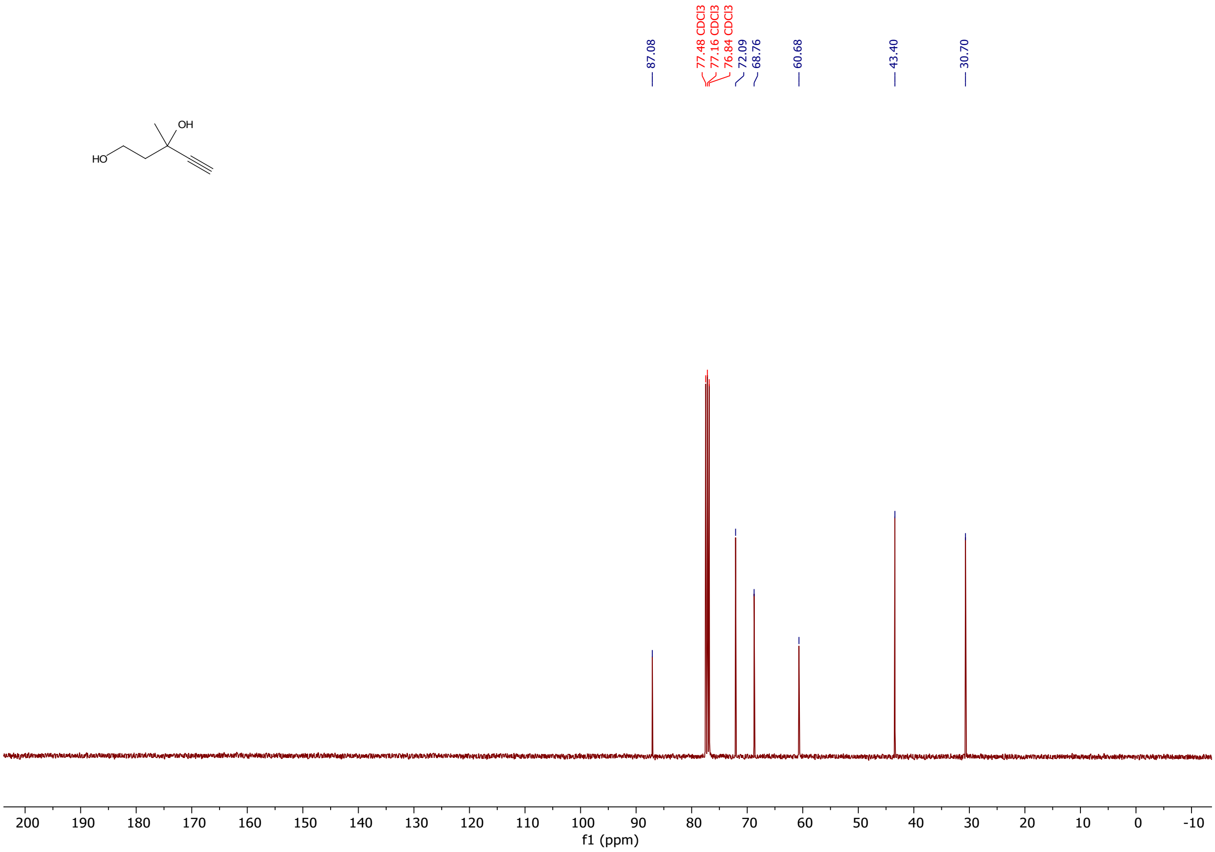

Figure 2: ^{13}C NMR spectrum of compound **6**.

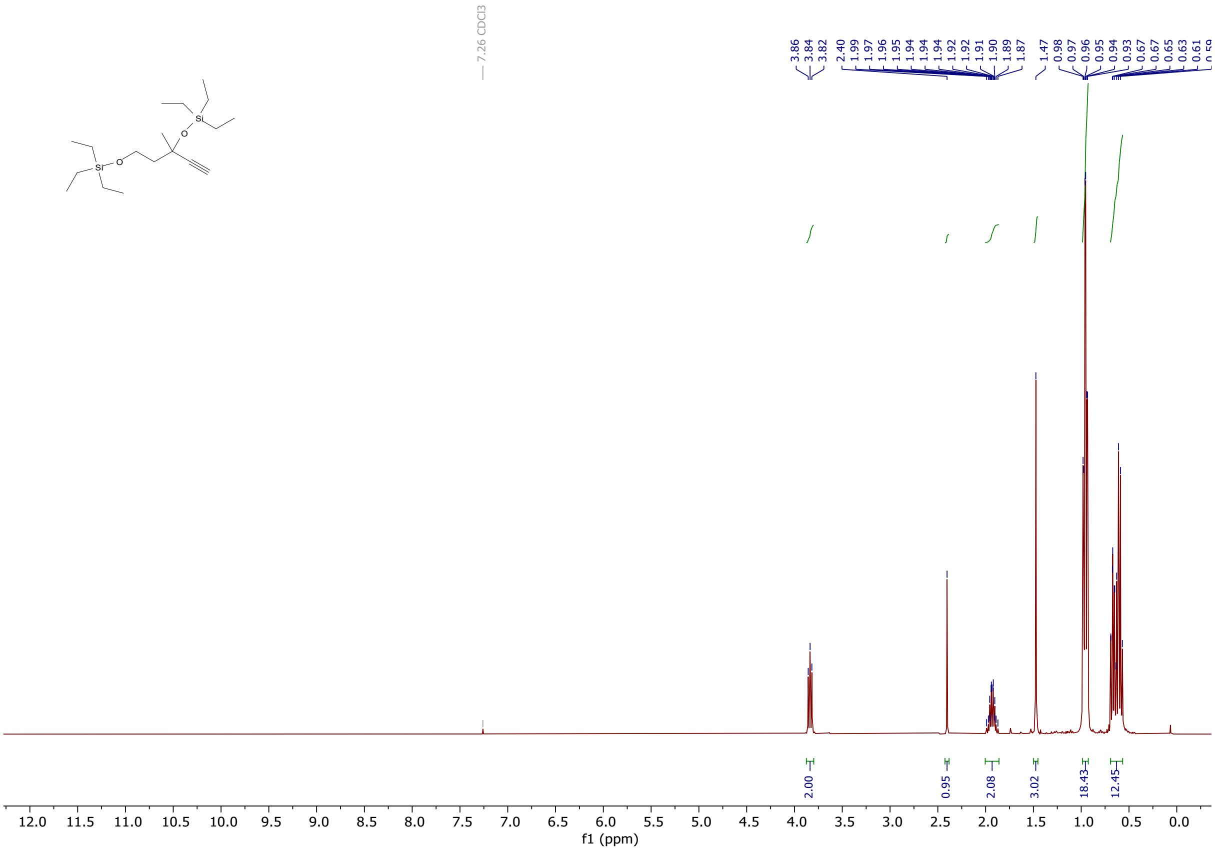

Figure 3: ^{1}H NMR spectrum of compound **7**.

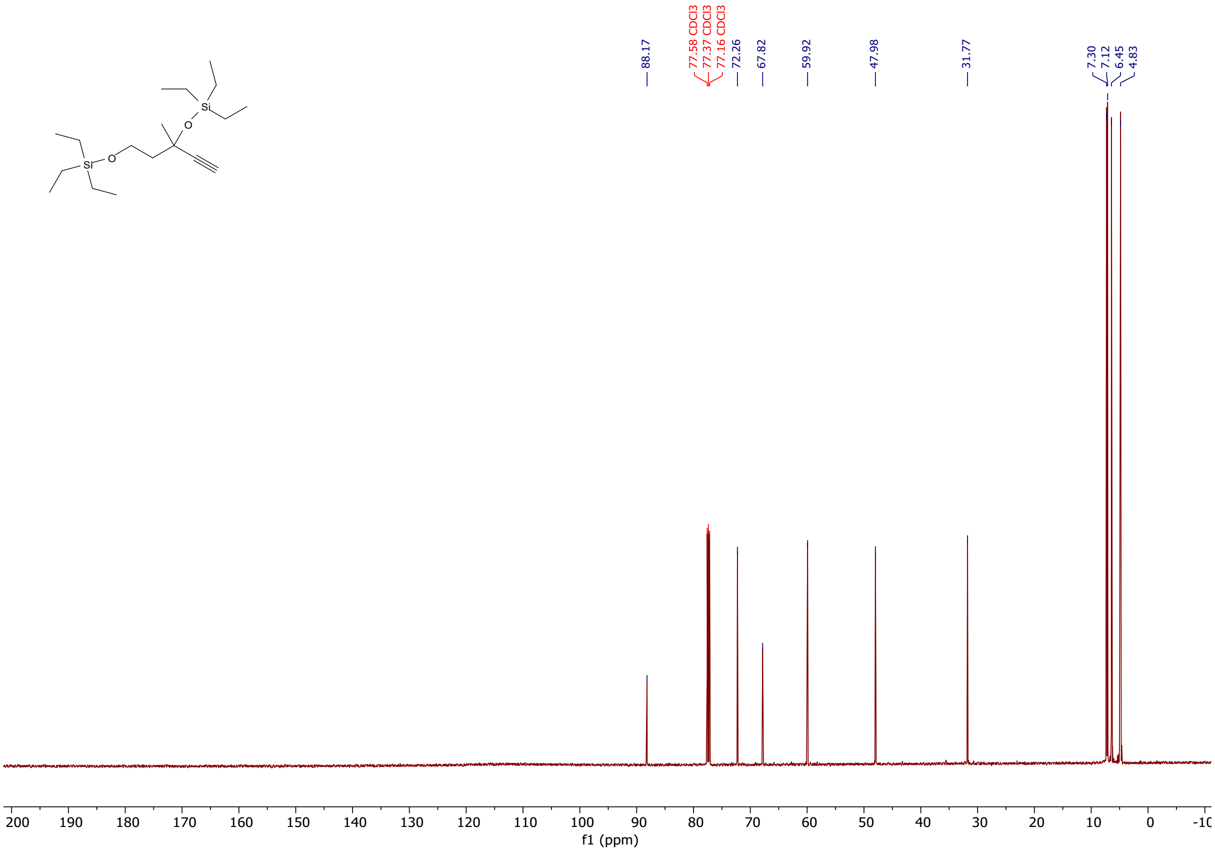

Figure 4: ^{13}C NMR spectrum of compound **7**.

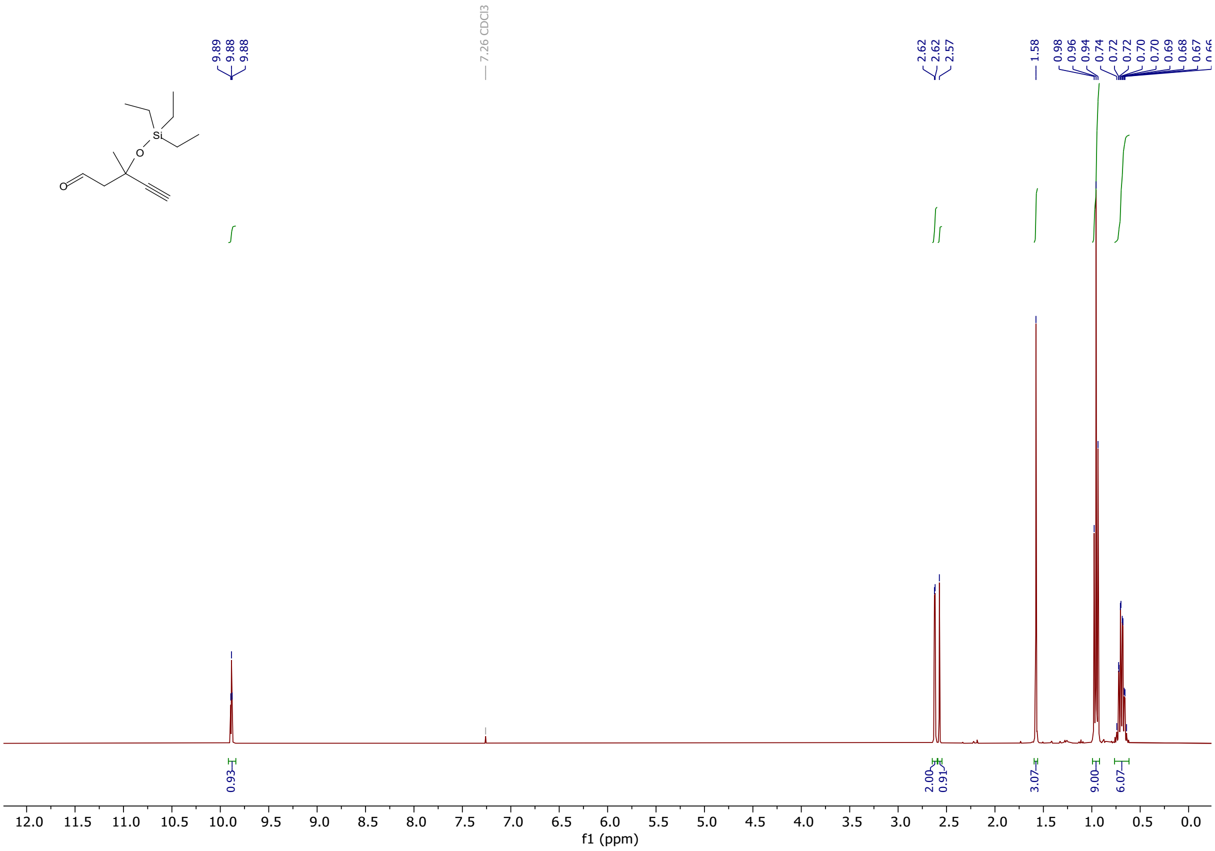

Figure 5: ^{1}H NMR spectrum of compound **8**.

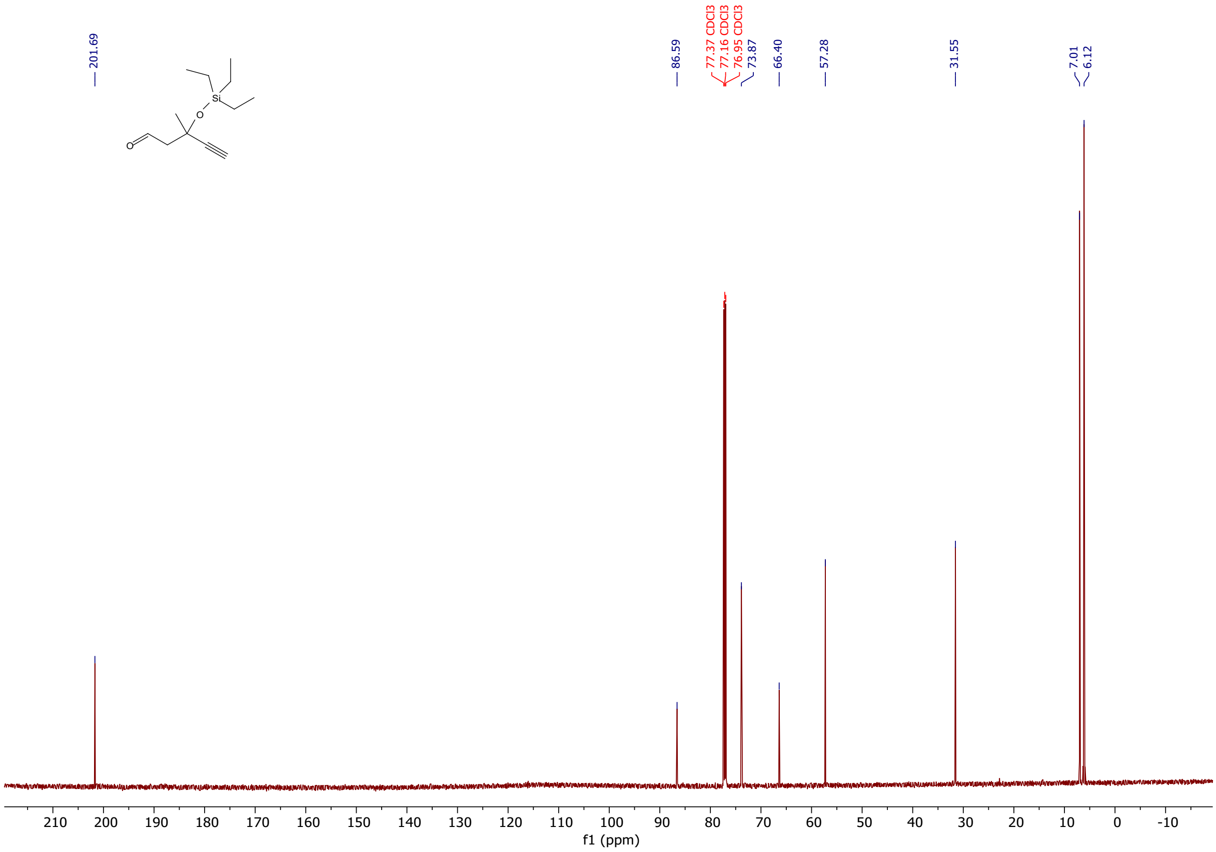

Figure 6: ^{13}C NMR spectrum of compound **8**.

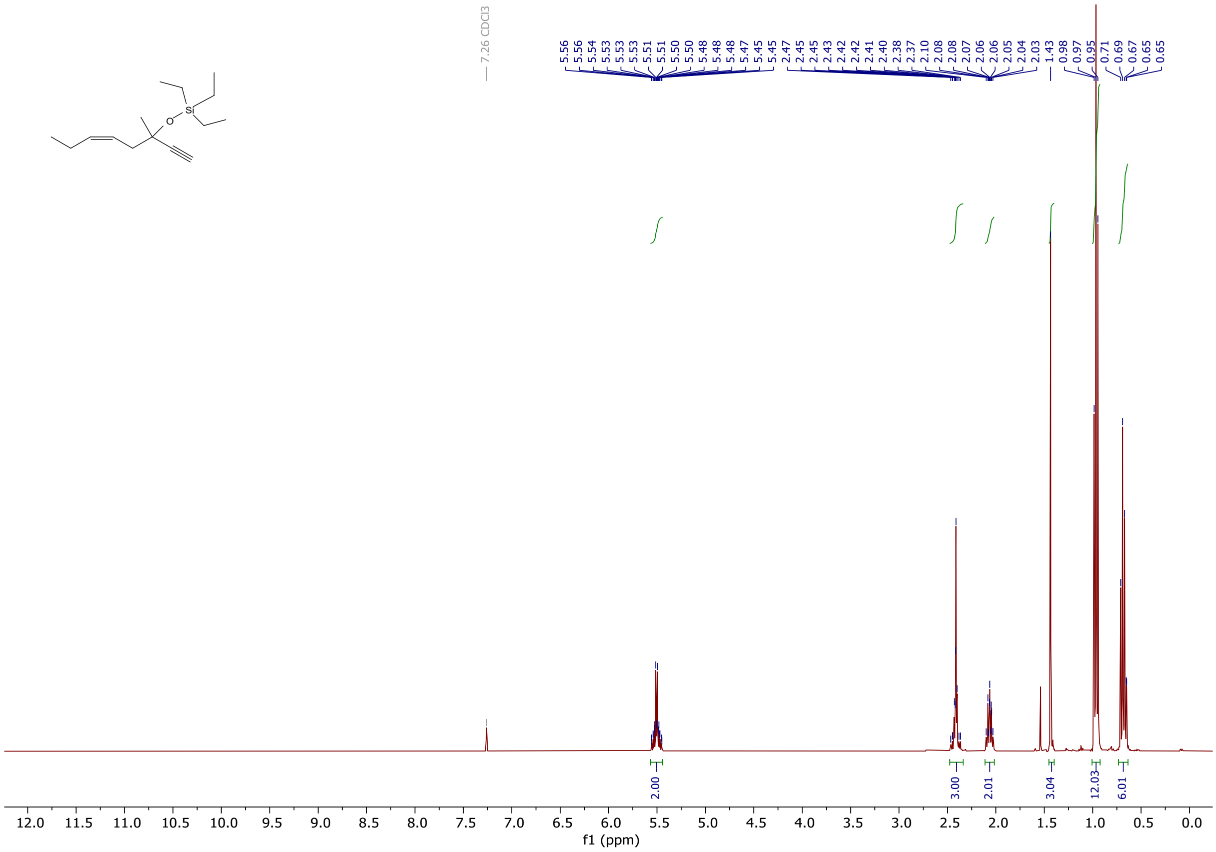

Figure 7: ^{1}H NMR spectrum of compound **9**.

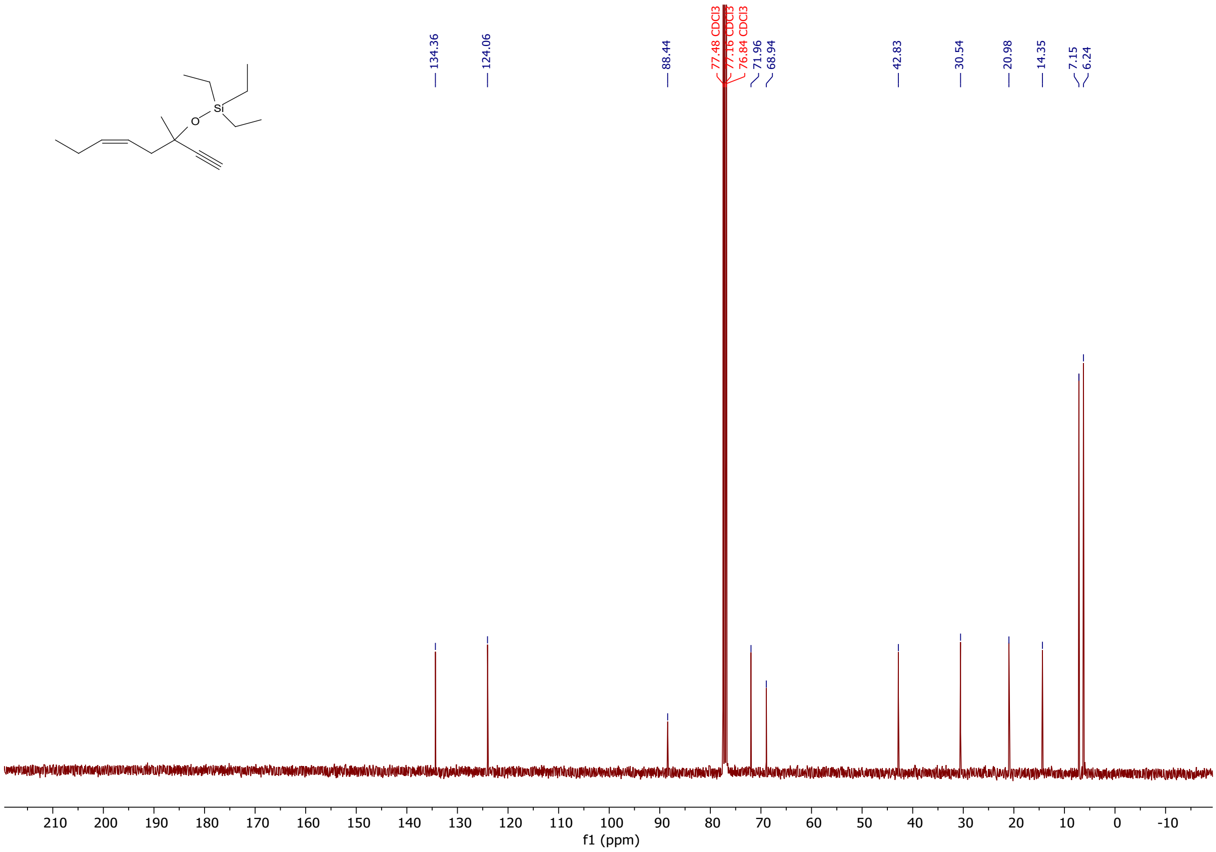

Figure 8: ^{13}C NMR spectrum of compound **9**.

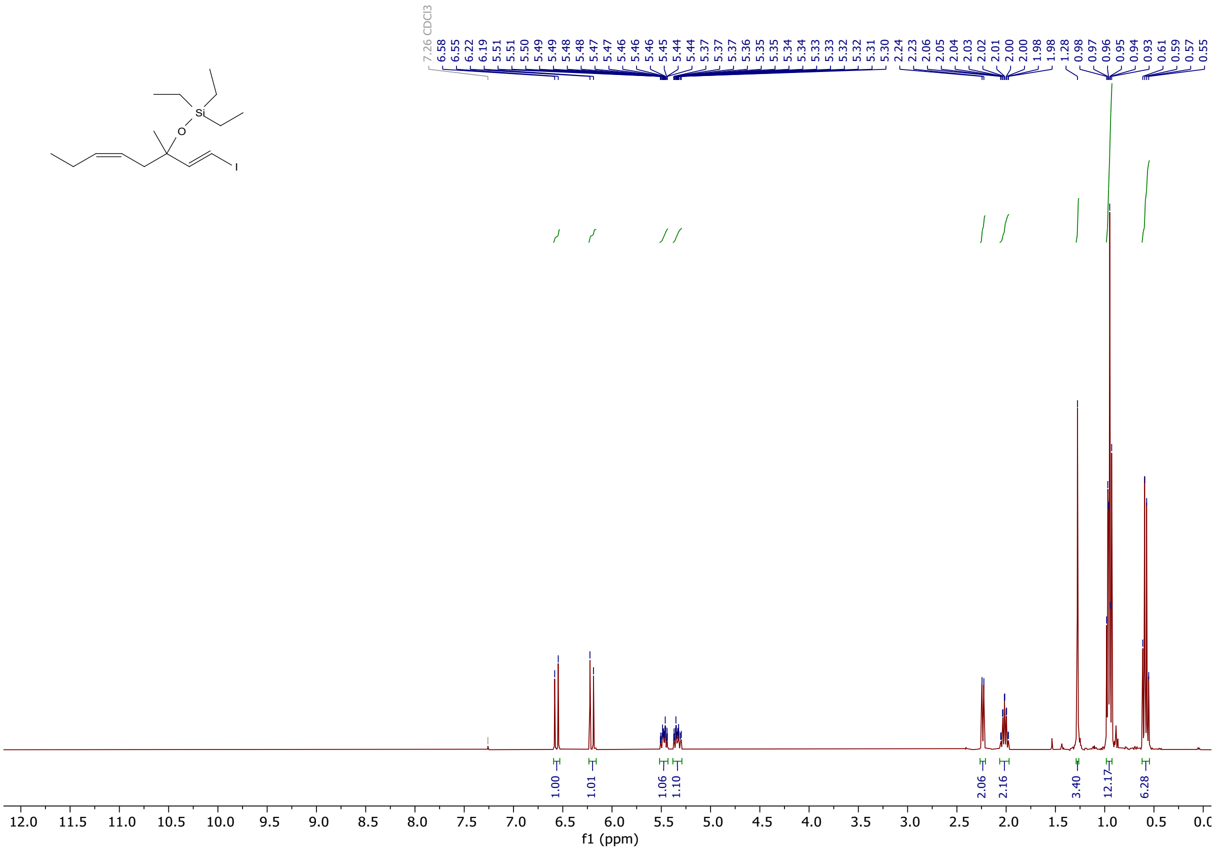

Figure 9: ^{1}H NMR spectrum of compound **3**.

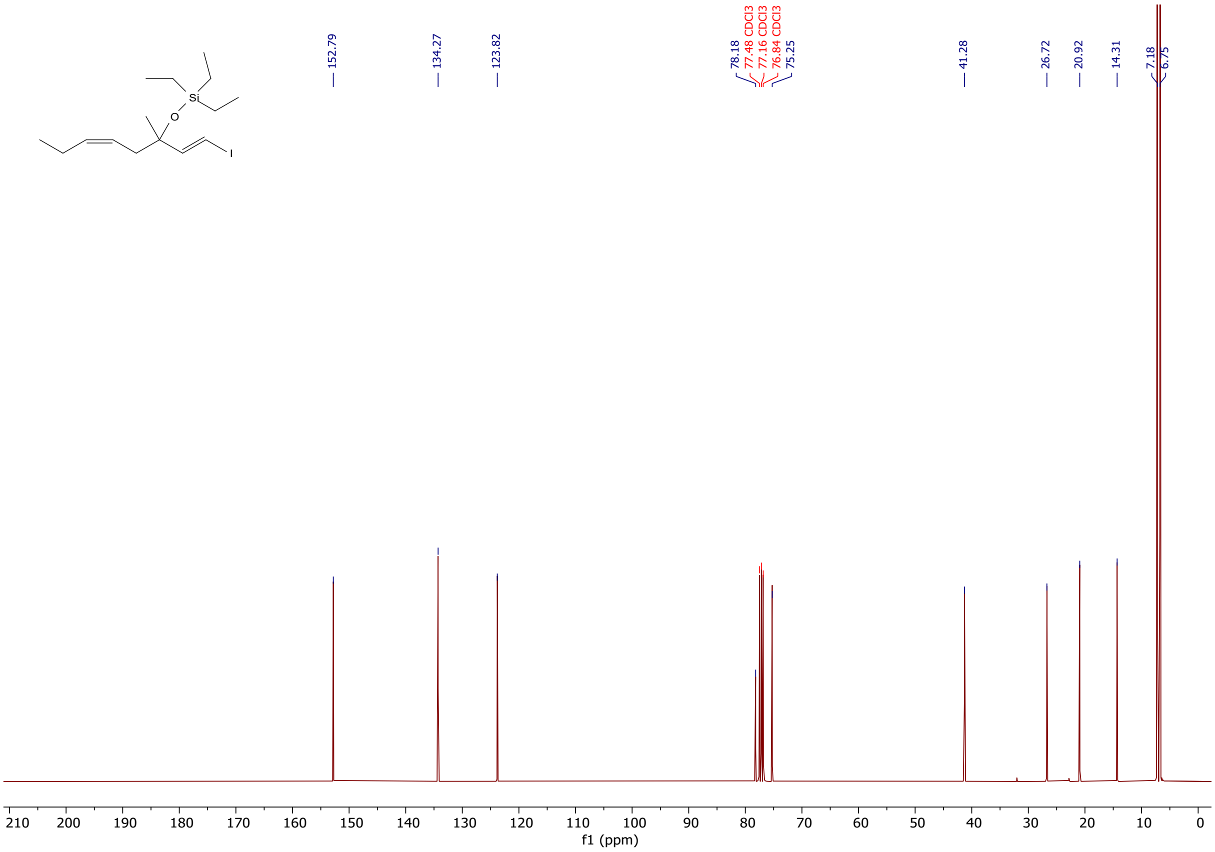

Figure 10: ^{13}C NMR spectrum of compound **3**.

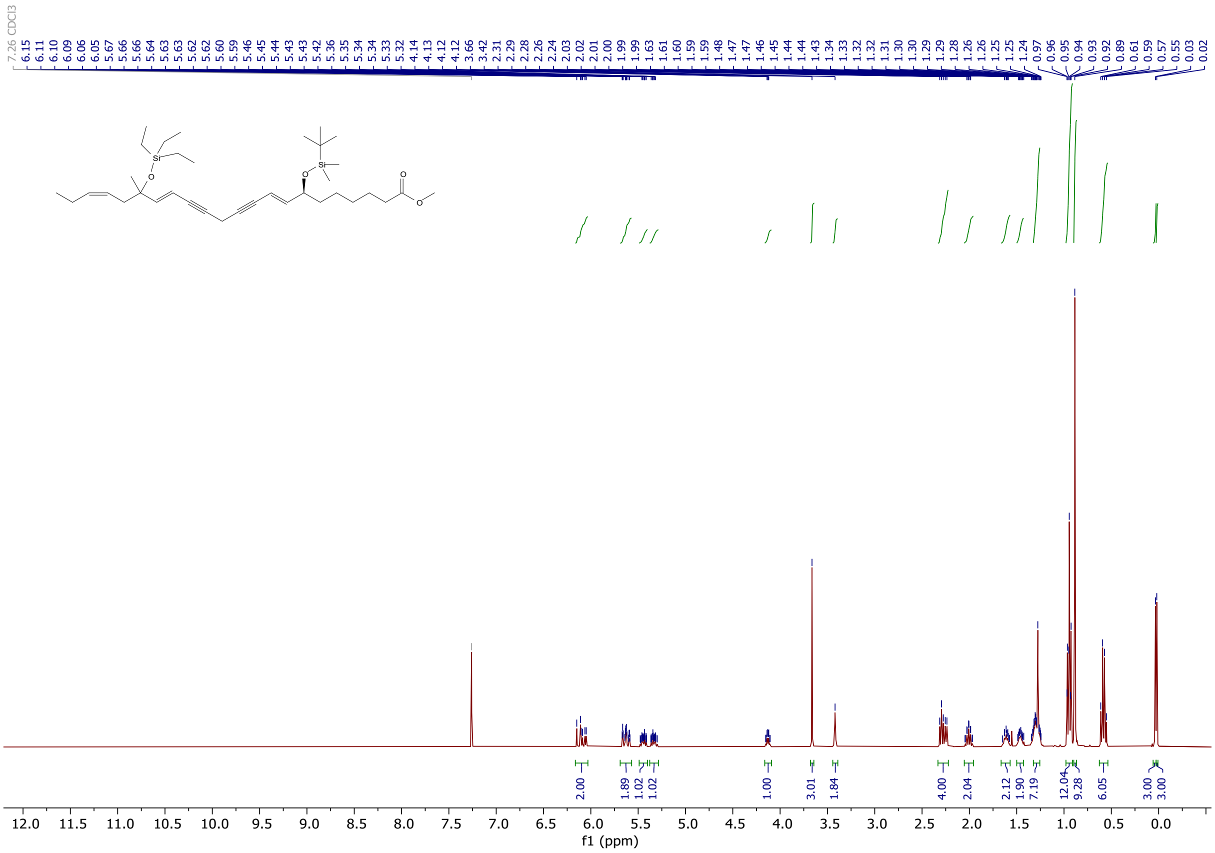

Figure 11: ^{1}H NMR spectrum of compound **10**.

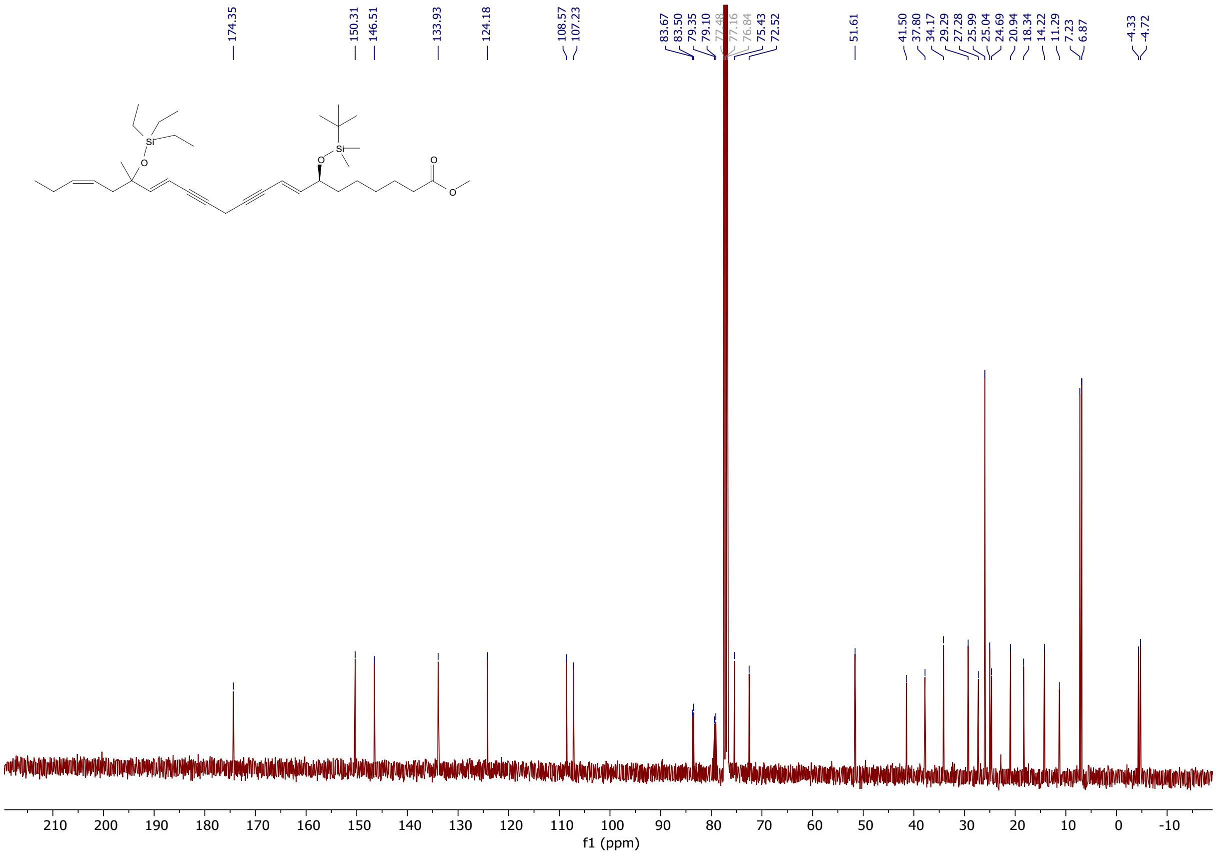

Figure 12: ^{13}C NMR spectrum of compound **10**.

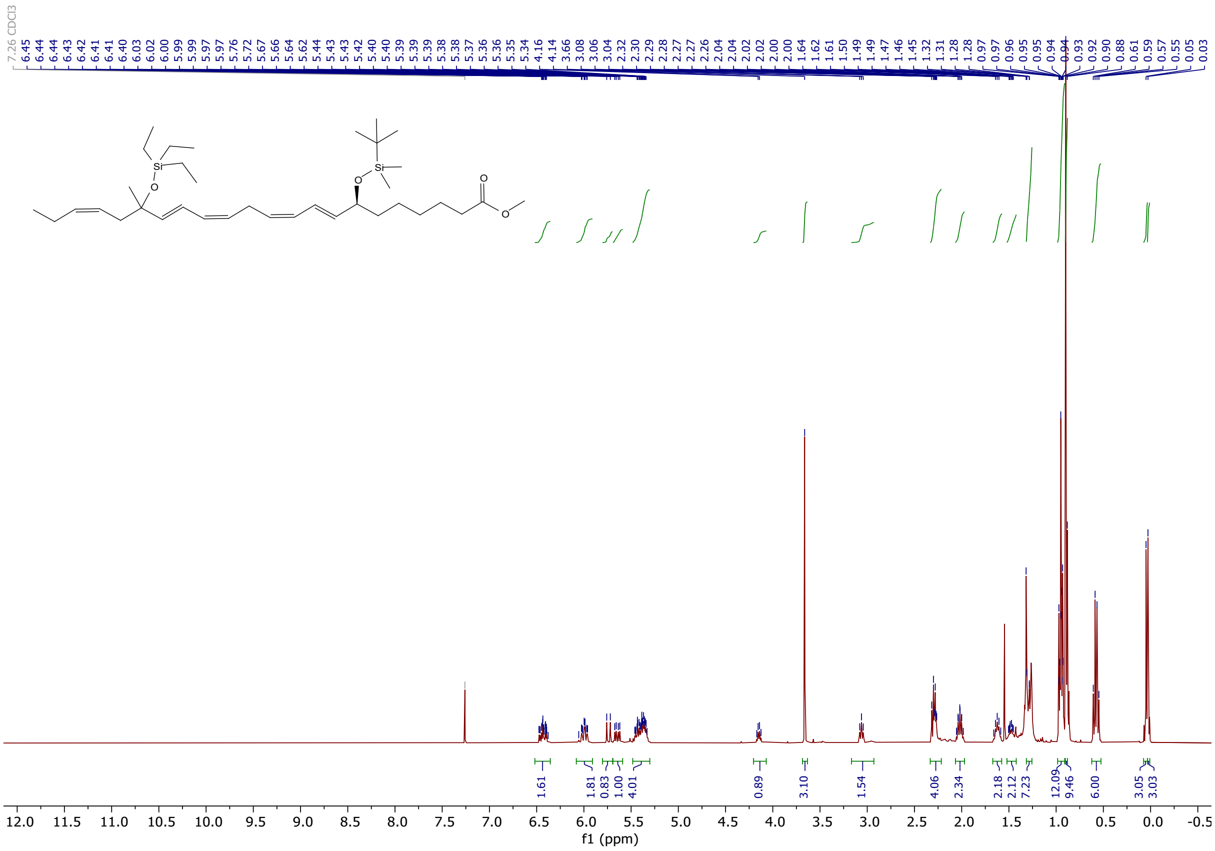

Figure 13: ^{1}H NMR spectrum of compound **11**.

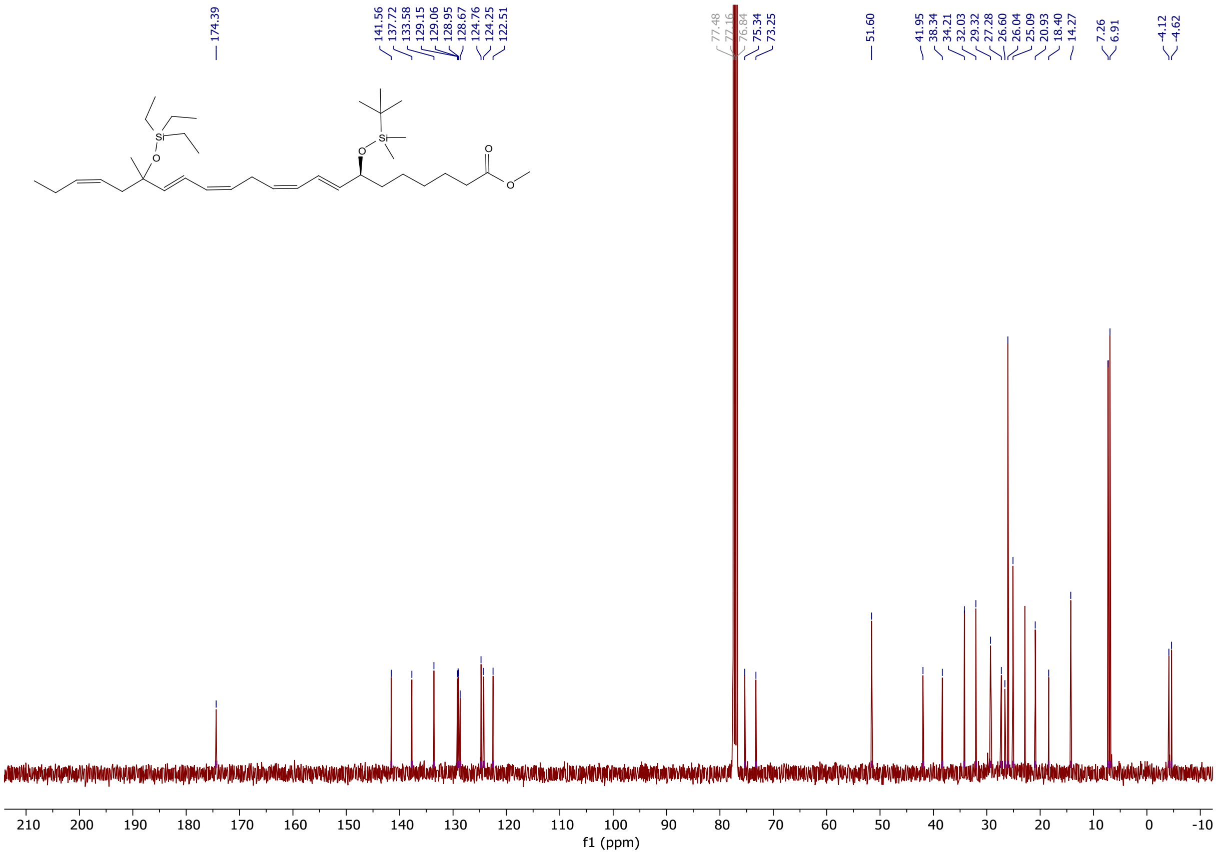

Figure 14: ^{13}C NMR spectrum of compound **11**.

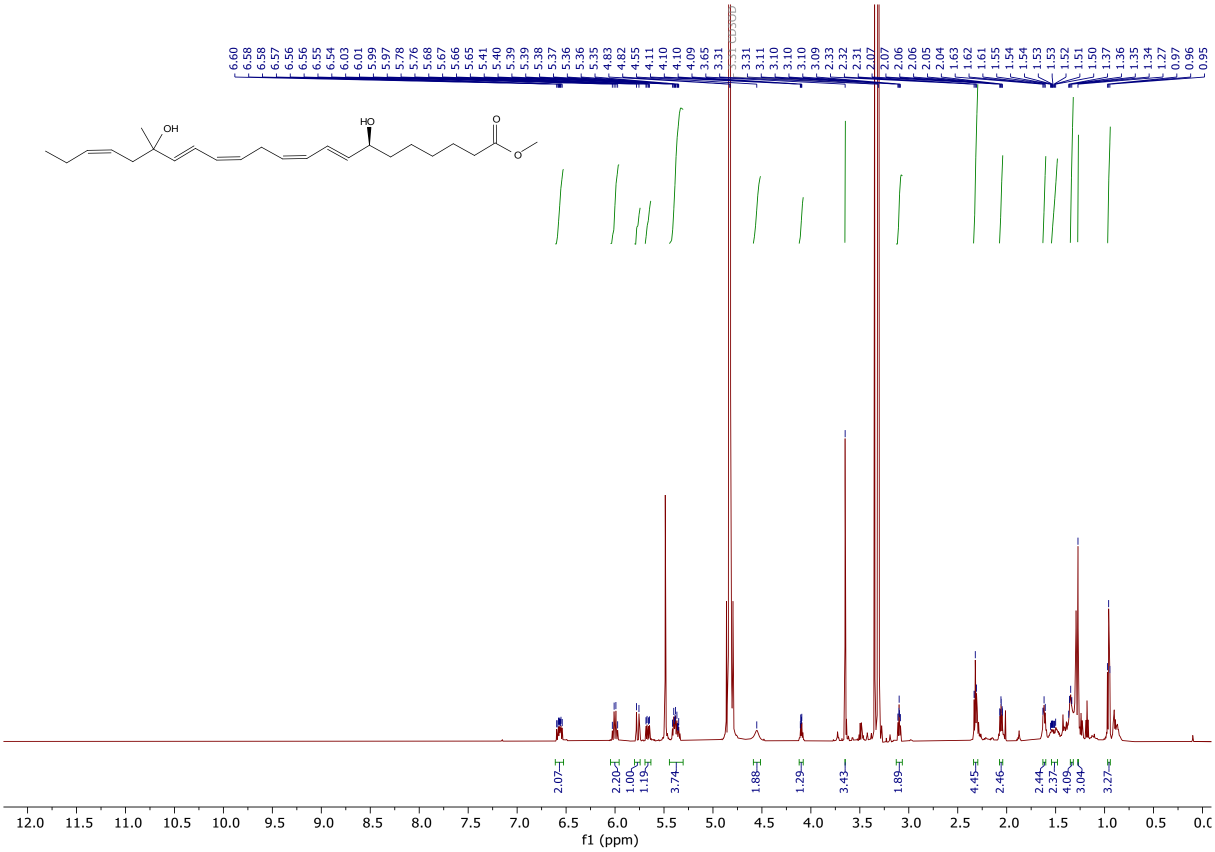

Figure 15: ^{1}H NMR spectrum of compound **12**.

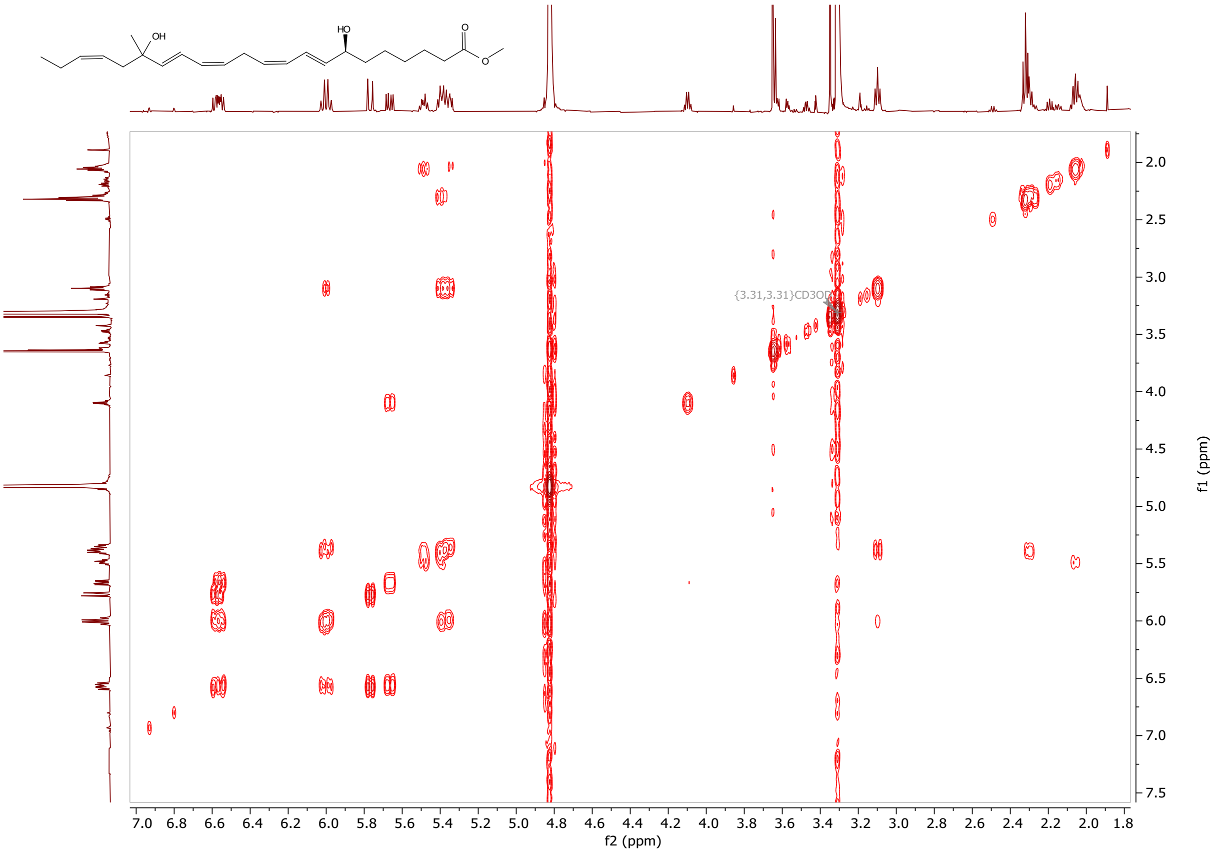

Figure 16: COSY NMR spectrum of compound **12**.

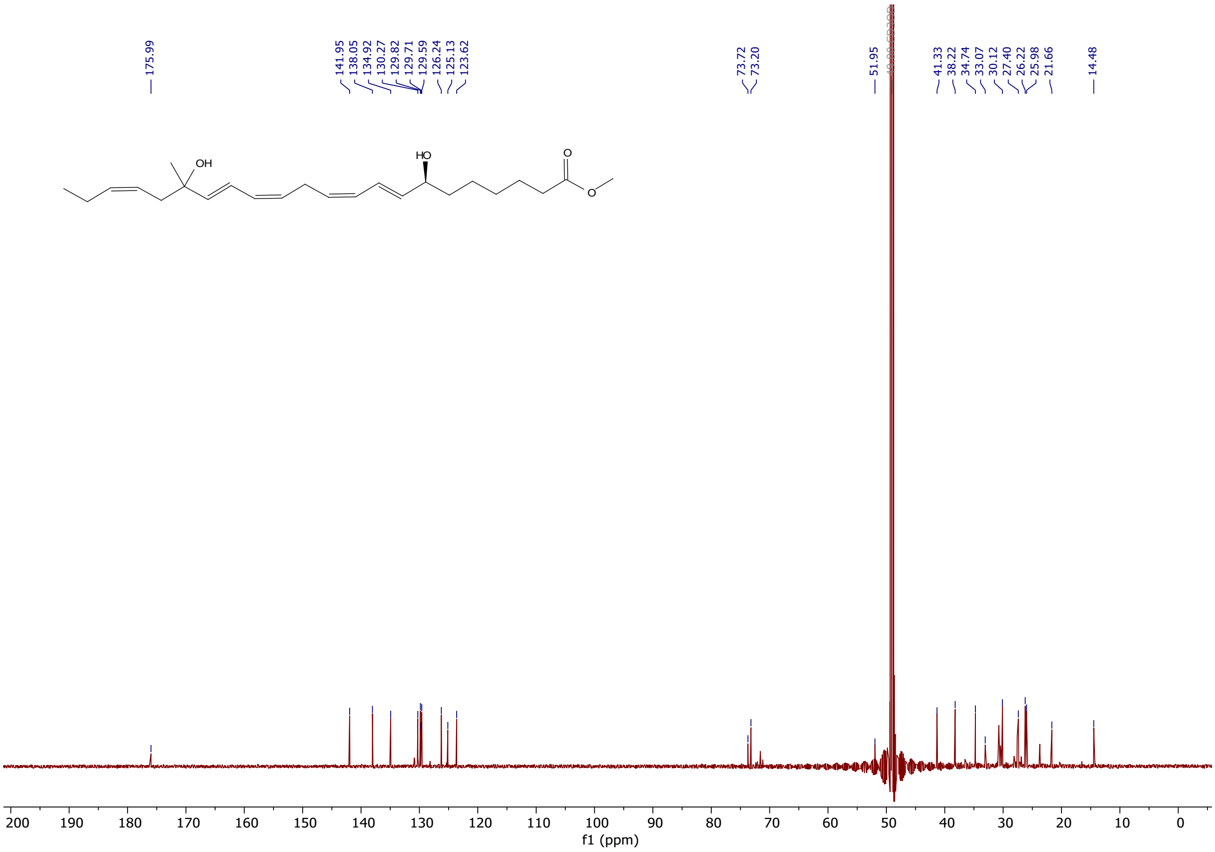

Figure 17: ^{13}C NMR spectrum of compound **12**.

HRMS Spectra

Elemental Analysis Report

**Analysis Info**			
Sample Name	KE144	Acquisition Date	17-Feb-23 1:13:45 PM
Method	ESI_pos_50_1500_os.m	Analysis Name	D:\Data\maxis2023\19310.d

Acquisition Parameter

Source Type	ESI	Set Capillary	3500 V	Set Nebulizer	0.5 Bar
Focus	Not active	Set End Plate Offset	-500 V	Set Dry Heater	200 °C
Scan Begin	50 m/z	Set Charging Voltage	2000 V	Set Dry Gas	4.0 l/min
Scan End	1500 m/z	Set Corona	0 nA	Set Divert Valve	Waste
				Set APCI Heater	0 °C

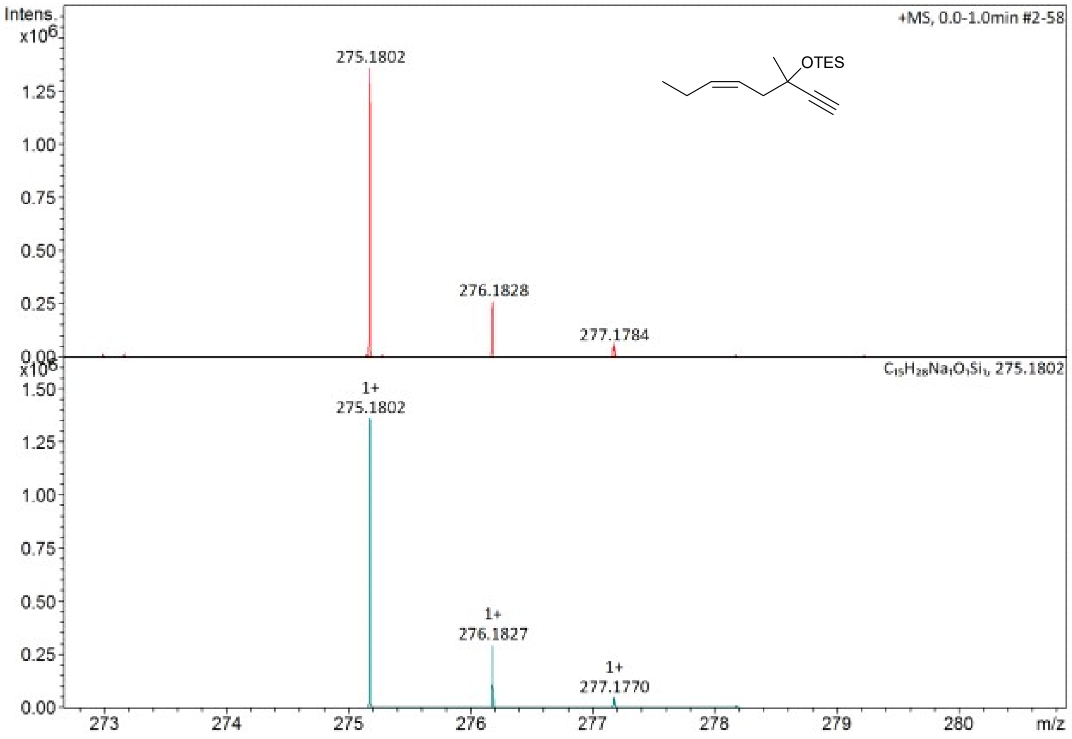

Meas. m/z	Ion Formula	m/z	err [ppm]
275.1802	C15H28NaOSi	275.1802	-0.1

Figure 18: HRMS spectrum of compound **9**.

Elemental Analysis Report

Analysis Info			
Sample Name	KE131	Acquisition Date	15-Dec-22 3:05:43 PM
Method	ESI_pos_50_1500_os.m	Analysis Name	D:\Data\maxis2022\19198.d

Acquisition Parameter

Source Type	ESI	Set Capillary	3500 V	Set Nebulizer	0.5 Bar
Focus	Not active	Set End Plate Offset	-500 V	Set Dry Heater	200 °C
Scan Begin	50 m/z	Set Charging Voltage	2000 V	Set Dry Gas	4.0 l/min
Scan End	1500 m/z	Set Corona	0 nA	Set Divert Valve	Waste
				Set APCI Heater	0 °C

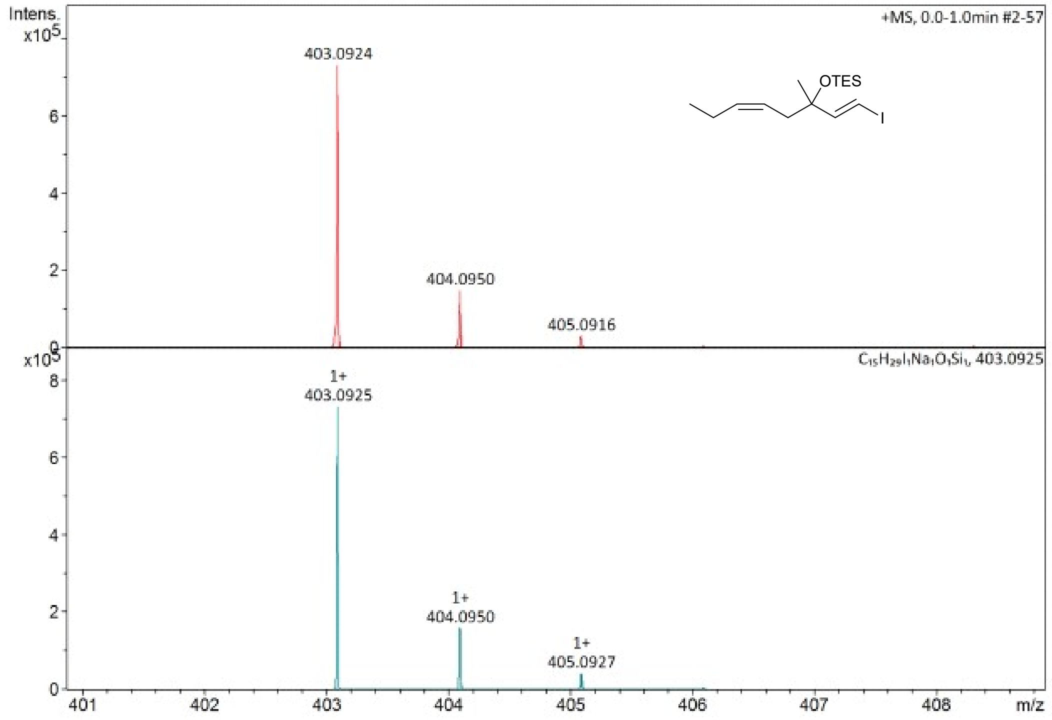

Meas. m/z	Ion Formula	m/z	err [ppm]
403.0924	C15H29INaOSi	403.0925	0.2

Figure 19: HRMS spectrum of compound **3**.

Elemental Analysis Report

Analysis Info

Sample Name	KE135	Acquisition Date	15-Dec-22 3:25:11 PM
Method	ESI_pos_50_1500_os.m	Analysis Name	D:\Data\maxis2022\19199.d

Acquisition Parameter

Source Type	ESI	Set Capillary	3500 V	Set Nebulizer	0.5 Bar
Focus	Not active	Set End Plate Offset	-500 V	Set Dry Heater	200 °C
Scan Begin	50 m/z	Set Charging Voltage	2000 V	Set Dry Gas	4.0 l/min
Scan End	1500 m/z	Set Corona	0 nA	Set Divert Valve	Waste
				Set APCI Heater	0 °C

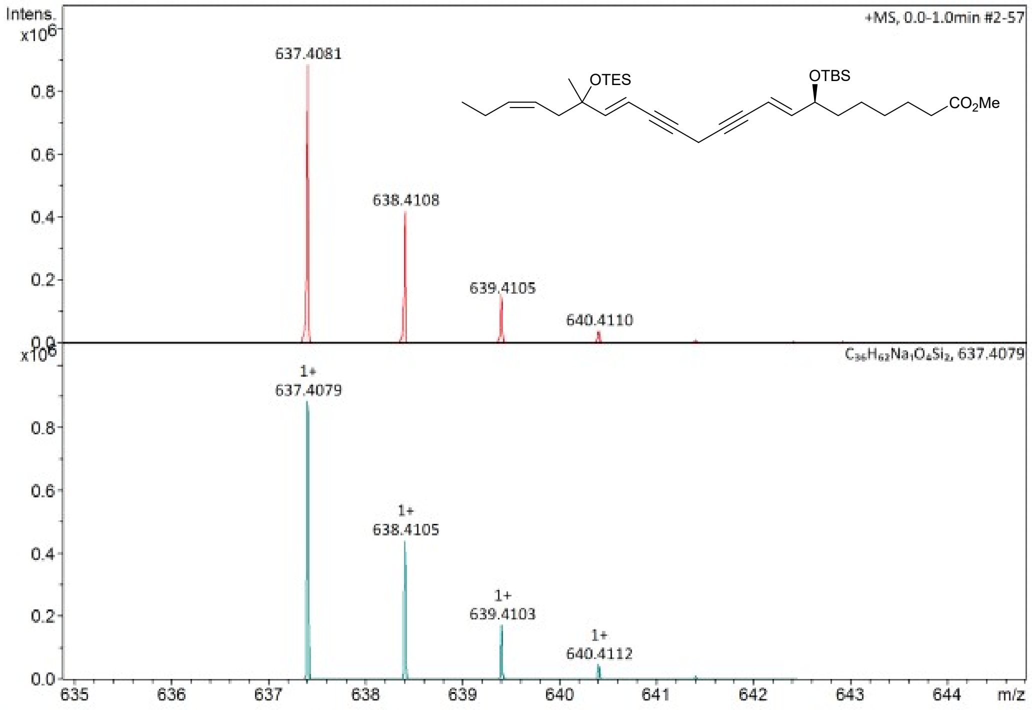

Meas. m/z	Ion Formula	m/z	err [ppm]
637.4081	C36H62NaO4Si2	637.4079	-0.4

Figure 20: HRMS spectrum of compound **10**.

Elemental Analysis Report

**Analysis Info**		Acquisition Date	03-Jan-23 3:58:24 PM
Sample Name	KE139.1	Analysis Name	D:\Data\maxis2022\19204.d
Method	ESI_pos_50_1500_os.m		

Acquisition Parameter

Source Type	ESI	Set Capillary	3500 V	Set Nebulizer	0.5 Bar
Focus	Not active	Set End Plate Offset	-500 V	Set Dry Heater	200 °C
Scan Begin	50 m/z	Set Charging Voltage	2000 V	Set Dry Gas	4.0 l/min
Scan End	1500 m/z	Set Corona	0 nA	Set Divert Valve	Waste
				Set APCI Heater	0 °C

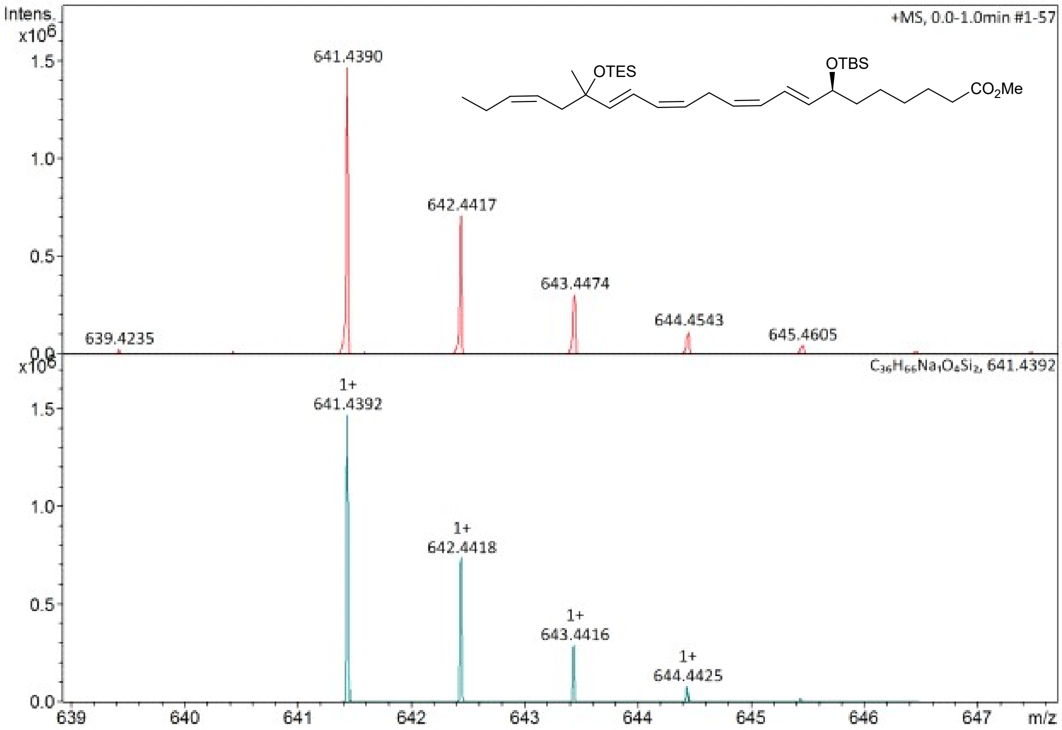

Meas. m/z	Ion Formula	m/z	err [ppm]
641.4390	C42H61O3Si	641.4384	-0.8
	C36H66NaO4Si2	641.4392	0.3

Figure 21: HRMS spectrum of compound **11**.

Elemental Analysis Report

Analysis Info

Sample Name	KE142	Acquisition Date	20-Jan-23 1:54:36 PM
Method	ESI_pos_50_1500_os.m	Analysis Name	D:\Data\maxis2023\19234.d

Acquisition Parameter

Source Type	ESI	Set Capillary	3500 V	Set Nebulizer	1.0 Bar
Focus	Not active	Set End Plate Offset	-500 V	Set Dry Heater	200 °C
Scan Begin	50 m/z	Set Charging Voltage	2000 V	Set Dry Gas	4.0 l/min
Scan End	1500 m/z	Set Corona	0 nA	Set Divert Valve	Waste
				Set APCI Heater	0 °C

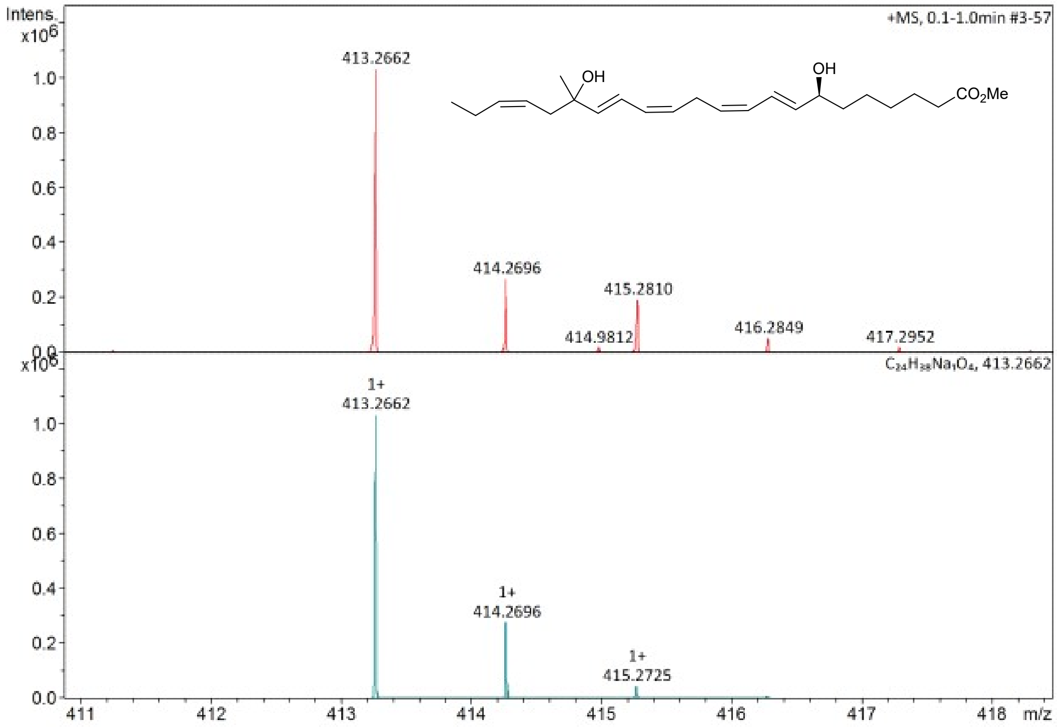

Meas. m/z	Ion Formula	m/z	err [ppm]
413.2662	C22H33N6O2	413.2660	-0.5
	C24H38NaO4	413.2662	0.2

Figure 22: HRMS spectrum of compound **12**.

HPLC chromatogram

Area Percent Report

Agilent Technologies

Data file: C:\Chem32\2\Data\Karina\KE142.3 2023-01-25 13-53-08.D
Instrument: LC1260-1
Description: 1 mL/min, C-18 MeOH/H2O 76/24, 250 nm, 10 uL injeksjonsvolum

**Sample name:**	KE142.3	**Injection date:**	1/25/2023 1:53:51 PM
**Sample type:**	Sample	**Acq. method:**	C-18SPM-Methylester.M
**Location:**	P1-A-01	**Analysis method:**	C-18SPM-Methylester.M
**Injection:**	1 of 1		
**Injection volume:**	10.000		

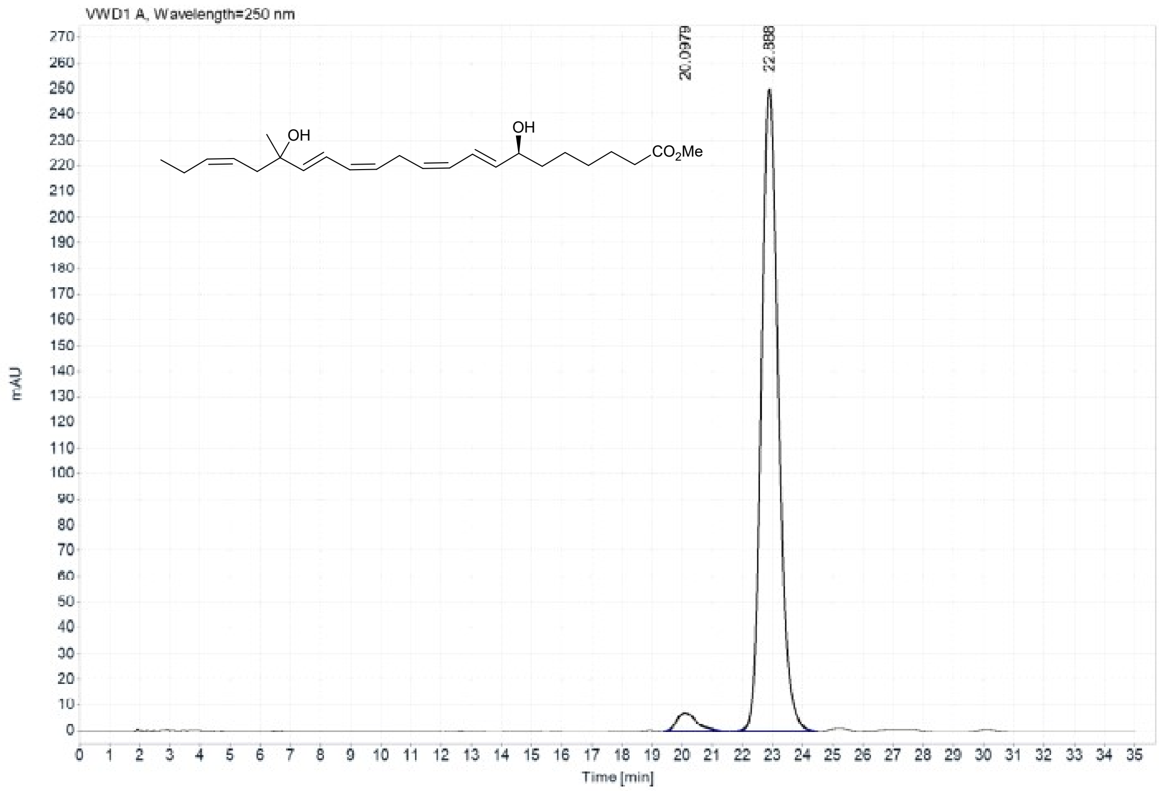

Signal: VWD1 A, Wavelength=250 nm

RT [min]	Type	Width [min]	Area	Height	Area%	Name
20.098	BB	0.7200	316.4789	6.7751	3.1313	
22.888	BB	0.6080	9790.4648	249.9353	96.8687	
		Sum	10106.9437			

Figure 23: HPLC chromatogram of compound **12**.

UV-Vis spectra

Hardcopy view   Date 1/25/2023 Time 13:54:24   Page   1 of 1

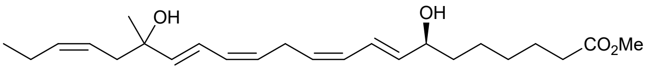

Overlaid Sample Spectra

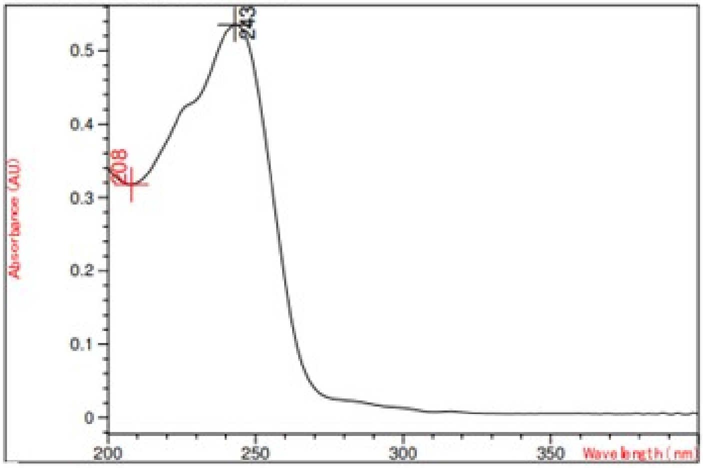

Sample/Result Table

#	Name	Peaks (nm)	Abs (AU)	Valleys (nm)	Abs (AU)
1		243.0	0.53536	208.0	0.31741
1		***	***	***	***
1		***	***	***	***

Figure 24: UV-Vis spectrum of compound **12**.

===

Hardcopy view   Date 3/13/2024 Time 14:25:32   Page   1 of 1

===

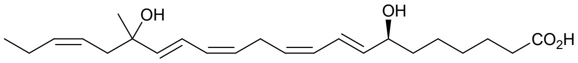

Last Sample Spectrum

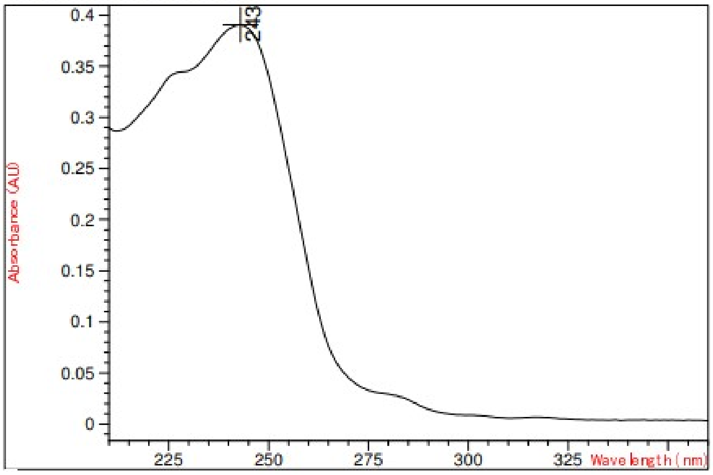

Sample/Result Table

#	Name	Peaks(nm)	Abs(AU)
1		243.0	0.39041

*** End Hardcopy view ***

Figure 25. UV-Vis spectrum of compound **2**.

References

1. J. Sueiras and W. H. Okamura, *J. Am. Chem. Soc.*, 1980, **102**, 6255-6259.
2. L. Zhang, N. Terrando, Z. Z. Xu, S. Bang, S. E. Jordt, W. Maixner, C. N. Serhan and R. R. Ji, *Front. Pharmacol.*, 2018, **9**, 412.
3. G. Chen, C. K. Park, R. G. Xie and R. R. Ji, *J. Clin. Invest.*, 2015, **125**, 3226-3240.
4. J. R. Deuis, L. S. Dvorakova and I. Vetter, *Front. Mol. Neurosci.*, 2017, **10**, 284.
